# Efficacy of metabarcoding for identification of fish eggs evaluated with mock communities

**DOI:** 10.1002/ece3.6144

**Published:** 2020-03-03

**Authors:** Elena M. Duke, Ronald S. Burton

**Affiliations:** ^1^ Marine Biology Research Division Scripps Institution of Oceanography University of California, San Diego La Jolla California

**Keywords:** 16S, cytochrome oxidase subunit I, fish egg surveys, Ichthyoplankton, metabarcoding

## Abstract

There is urgent need for effective and efficient monitoring of marine fish populations. Monitoring eggs and larval fish may be more informative than that traditional fish surveys since ichthyoplankton surveys reveal the reproductive activities of fish populations, which directly impact their population trajectories. Ichthyoplankton surveys have turned to molecular methods (DNA barcoding & metabarcoding) for identification of eggs and larval fish due to challenges of morphological identification. In this study, we examine the effectiveness of using metabarcoding methods on mock communities of known fish egg DNA. We constructed six mock communities with known ratios of species. In addition, we analyzed two samples from a large field collection of fish eggs and compared metabarcoding results with traditional DNA barcoding results. We examine the ability of our metabarcoding methods to detect species and relative proportion of species identified in each mock community. We found that our metabarcoding methods were able to detect species at very low input proportions; however, levels of successful detection depended on the markers used in amplification, suggesting that the use of multiple markers is desirable. Variability in our quantitative results may result from amplification bias as well as interspecific variation in mitochondrial DNA copy number. Our results demonstrate that there remain significant challenges to using metabarcoding for estimating proportional species composition; however, the results provide important insights into understanding how to interpret metabarcoding data. This study will aid in the continuing development of efficient molecular methods of biological monitoring for fisheries management.

## Graphical abstract

We examine the effectiveness of using metabarcoding methods on mock communities of known fish egg DNA using six mock communities with known ratios of species and two samples from a large field collection of fish eggs. We examine the ability of our metabarcoding methods to detect species and relative proportion of species identified in each mock community. We found that our metabarcoding methods were able to detect species at very low input proportions; however, levels of successful detection depend on the markers used in amplification suggesting that the use of multiple markers is desirable.

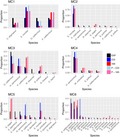

## 1 INTRODUCTION

Marine ecosystems can change rapidly in response to both natural and anthropogenic changes in the environment. Monitoring changes in the abundance and distribution of marine organisms is a challenging but critical component of conservation efforts and natural resource management. Human impacts such as overfishing, coastal development, and pollution can rapidly increase stress on ecosystems resulting in an urgent need to develop efficient methods for monitoring spatial and temporal dynamics of biodiversity (Beng et al., 2016).

Fisheries management has long used ichthyoplankton surveys as a component of monitoring because sampling the early life stages of fish reflects the reproductive activities of fish populations, a sensitive indicator of population growth or decline (Alstrom & Moser, 1976; Koslow, Miller, & McGowan, 2015). Early life stages of fish including eggs and larvae have the advantage of being easily sampled in plankton tows, rather than logistically difficult visual observation surveys or invasive methods such as trawls and seines (Craig, Fodrie, & Hastings, 2004; Harada et al., 2015; Thomsen et al., 2012). However, a significant problem with plankton analyses is that morphological identification of ichthyoplankton is difficult for even well‐trained scientists and errant identifications are frequent enough to make quantitative conclusions problematic. This problem has been addressed by moving from morphological identifications to the application of “DNA barcoding” where a small fragment of DNA is amplified from individual fish eggs by PCR and then sequenced. The resulting sequences are compared to existing databases for species identification. However, for situations involving the identification of large numbers of eggs, the sequencing of individual fish eggs is both time‐consuming and expensive.

Metabarcoding provides an alternative to traditional barcoding that involves analysis of pooled samples of individuals (potentially belonging to many species) using next‐gen sequencing (NGS) (Cristescu, 2014). Indeed, metabarcoding has long been applied to microbial communities where analysis of single individual cells is often impractical (Egge et al., 2013; Klindworth et al., 2013; Gilbert, Jansson, & Knight, 2014; Tringe et al., 2005; Venter et al., 2004), and this approach is gaining traction for species identification in bulk DNA samples for biodiversity studies of larger eukaryotes (Ficetola, Miaud, Pompanon, & Taberlet, 2008; Kelly, Port, Yamahara, & Crowder, 2014; Miya et al., 2015; Thomsen et al., 2012). Metabarcoding has the potential to increase speed and efficiency of biological monitoring, which may contribute to our understanding of many ecological issues including species‐level discrimination, community composition, and early detection of invasive, cryptic, rare, or threatened species (Bik et al., 2012; Cristescu, 2014; Ficetola et al., 2008; Miya et al., 2015).

Relatively few studies have assessed the quantitative accuracy of metabarcoding results for diverse DNA samples. For example, it has generally been assumed that the proportion of reads obtained for a given species is proportional to the contribution of DNA from that species within the sample relative to DNA from other taxa (Amend, Seifert, & Bruns, 2010; Egge et al., 2013). However, it is well documented that an intermediate PCR step produces amplification bias and can influence the accuracy of metabarcoding results and subsequent biodiversity estimates (Bik et al., 2012). Moreover, target genes used for species identification are located on the mitochondrial DNA (mtDNA) and the copy number of mtDNA across species and even tissue type can impact the accuracy of biomass estimates (Hatzenbuhler, Kelly, Martinson, Okum, & Pilgrim, 2017; Schloss, Gevers, & Westcott, 2011). Over‐ or underestimating biodiversity from NGS data can have detrimental managerial consequences and underscores the need for experimental validation of metabarcoding protocols (Amend et al., 2010; Cristescu, 2014).

In the current study, we examine the efficacy of metabarcoding protocols on mock communities created with known ratios of input fish eggs and on a natural community that was simultaneously analyzed by traditional barcoding. We constructed six mock communities of at least 100 fish eggs that were previously sequenced and identified using DNA barcoding. Additionally, we analyzed two sets of 500 unknown fish eggs from a natural collection to compare to a large sample (*n* = 1,000) of individually identified eggs from the same natural collection. We used the Illumina MiSeq platform to perform metabarcoding with sequences of cytochrome oxidase subunit I (COI), and mitochondrial 16S ribosomal RNA both separately and in combination to examine the extent to which next‐gen sequencing results can (a) detect presence of species in mixed samples and (b) identify the relative proportions of species in a community. These data are critical for understanding how to interpret and draw conclusions from metabarcoding data used in biological monitoring and fisheries management.

## 2 MATERIALS AND METHODS

### 2.1 Sampling locations and techniques

Weekly plankton samples were collected from two sites. (a) Plankton samples were collected from the end of Scripps Pier (32.8328°N, −117.2713°W) from August 2014 to August 2017. Samples were collected by lowering a 505‐micron mesh one‐meter‐diameter plankton net until the net reached the seafloor around midday each sampling day. This was repeated three more times for a total of four pulls, sampling a total of approximately 16 cubic meters of water (based on average water depth of about 5 m). (b) Weekly plankton samples were collected from kelp forest habitat adjacent to the Matlahuayl Marine Reserve (32.85408°N, 117.28105°W) from February 2017 to August 2017. These latter samples were collected by pulling a 333‐micron mesh one half‐meter‐diameter plankton net behind a small boat at 0.5 knots for 5 min. The net was weighted for a sampling depth of about 1 m, sampling approximately 60 cubic meters of water. The collected plankton samples from both sites were manually sorted using a dissecting microscope, and fish eggs were individually counted and removed. The Northern Anchovy (*Engraulis mordax*) and Pacific sardine eggs (*Sardinops sagax*), both morphologically distinct, were counted and removed from the sample. The remaining eggs were stored in 95% ethanol at 4°C for at least 12 hr prior to further processing.

### 2.2 Identification of individual fish eggs

Individual fish eggs were rinsed with molecular‐grade water and placed in 15 μl of buffer (2/3 Qiagen AE buffer, 1/3 molecular‐grade water). Eggs were then physically squished with a clean pipette tip to release the DNA. No further DNA extraction or purification was necessary. Samples were stored at −20°C prior to polymerase chain reaction (PCR). To amplify DNA, we used universal fish cytochrome *c* oxidase subunit I (COI) primers (Ward, Zemlak, Innes, Last, & Hebert, 2005): COI VF1 forward primer (5′‐TTCTCAACCAACCACAAAGACATTGG‐3′) and COI VR1 reverse (5′‐TAGACTTCTGGGTGGCCAAAGAATCA‐3′). These markers were selected to take advantage of the near‐complete COI and 16S barcoding databases (in NCBI's GenBank) available for California marine fish produced in large part by the Burton laboratory in collaboration with the Scripps Institution of Oceanography's Marine Vertebrate Collection. Sequences for each fish species were obtained with these primers in our laboratory, so we have some confidence that amplification of the target genes will be successful in the local species. The COI primers produced an amplicon of 710 bp. PCR was performed using 25 μl reaction volume, with 12.5 μl of GoTaq Green Master Mix (Promega), 5 pmol of each primer, and 1 μl of DNA extract (Hajibabaei et al., 2005). Thermal cycling was initiated at 95°C for 2 min followed by 35 cycles of 95°C for 30 s, 50°C for 45 s, and 72°C for 1 min, followed by 72°C for 5 min. After PCR, samples were run on a 1.5% agarose gel and visualized with GelRed (Biotium) or SybrSafe (Invitrogen) to detect presence of amplified DNA. When samples failed to amplify with COI, amplification of the mitochondrial 16S ribosomal rRNA gene was attempted using forward primer 16Sar (5′‐CGCCTGTTATCAAAAACAT‐3′) and reverse primer 16Sbr (5′‐CCGGTCTGAACTCAGATCACGT‐3′) for a 570 bp amplicon **(**Palumbi, 1996). COI or 16S products were purified using Sephadex G‐50 Fine spin columns and sequenced using Sanger sequencing (commercial sequencing service). We note that a shorter amplicon of the 12S gene is a frequent target for metabarcoding and eDNA studies, but many of the species found in our study region do not have published sequences in the NCBI database for the 12S marker. In addition, the 12S gene does not provide the same level of taxonomic resolution as COI or 16S. Amplified sequences were identified using BLAST searches of NCBI database, which contains DNA barcodes from over 600 species of California marine fishes most of which are vouchered in the Scripps Institution of Oceanography Marine Vertebrate Collection, allowing for nearly complete coverage of species in California marine waters (Hastings & Burton, 2008). The top BLAST hit with 97% sequence similarity or greater was used for species identification (Hatzenbuhler et al., 2017; Miya et al., 2015). Given that we have extensive data on fish eggs in the study area, the 97% criterion can be used to distinguish essentially all eggs for which we obtain 150 bp of clean sequence (Duke, Harada, & Burton, 2018; Harada et al., 2015). This level of stringency allows for the range of Taq polymerase error and Illumina sequencing error intrinsic to our methodology.

### 2.3 Mock community construction

Six mock communities were constructed by combining the extracts of individual fish eggs (i.e., the remainder of the squished fish egg and Qiagen AE buffer) that had been previously identified. Each sample consisted of at least 100 eggs representing between 4 and 17 species in either equal or skewed proportions (Table 1). After the desired combination of eggs had been combined into a single 2‐ml tube, each mock community was homogenized using a Mini‐Beadbeater with 0.5 mm beads (BioSpec products) and vortexed. Half of the mixture was then extracted using the Qiagen DNeasy Blood and Tissue Kit and the other half saved and stored at −20°C. Mock communities were amplified using three primer combinations: (a) universal fish cytochrome *c* oxidase subunit I (COI) primers, (b) mitochondrial 16S ribosomal rRNA gene (16S), and (c) multiplexed both COI and 16S primers in a single reaction. Again, these markers were chosen to take advantage of the near‐complete barcode database for California marine fish using these genes. Mock communities were amplified using the following PCR protocol: PCR was performed using 25 μl reaction volume, with 12.5 μl of GoTaq Green Master Mix, 5 pmol of each primer, and 1 μl of DNA extract. When the PCR was multiplexed (simultaneous use of the two primer pairs), the final concentration of each primer was 5 pmol and 2.5 pmol for COI and 16S, respectively. GoTaq was selected because we high a high success rate identifying individual eggs using this master mix; although it lacks 3′→5′ exonuclease activity (i.e., proofreading), the relatively rare (10^–5^) error rate is unlikely to cause any problems given our species identification criterion of 97% identity. Thermal cycling was initiated at 95°C for 2 min followed by 25 or 30 cycles of 95°C for 30 s, 50°C for 45 s, and 72°C for 1 min, followed by 72°C for 5 min. After PCR, samples were run on a 1.5% agarose gel and visualized with GelRed or SybrSafe to detect presence of amplified DNA. We used 25 amplification cycles in some of our PCRs in an effort to reduce amplification bias (Pawluczyk et al., 2015; Schloss et al., 2011).

**Table 1:** List of species that constituted six mock communities and their proportion in each mock community

Species	MC1	MC2	MC3	MC4	MC5	MC6
*Anisotremus davidsonii*	0	0	0	0	0.050	0.02
*Cheilotrema saturnum*	0	0	0	0	0	0.01
*Chilara taylori*	0	0	0.010	0	0	0
*Citharichthys stigmaeus*	0.25	0	0	0	0.010	0
*Cynoscion parvipinnis*	0	0	0	0	0	0.04
*Engraulis mordax*	0.25	0.571	0	0	0.050	0
*Etrumeus acuminatus*	0	0	0	0	0	0.01
*Genyonemus lineatus*	0	0	0.030	0	0	0.02
*Halichoeres semicinctus*	0	0.057	0.168	0.7	0.099	0
*Hippoglossina stomata*	0	0	0	0	0	0.01
*Menticirrhus undulatus*	0	0	0	0.1	0.050	0
*Oxyjulis californica*	0.25	0	0.495	0	0	0
*Paralabrax clathratus*	0	0.086	0	0	0	0.03
*Paralichthys californicus*	0.25	0.029	0.149	0	0.099	0.2
*Pleuronichthys coenosus*	0	0	0	0	0	0.01
*Pleuronichthys verticalis*	0	0	0	0	0	0.02
*Roncador stearnsii*	0	0.057	0.099	0	0	0.03
*Sardinops sagax*	0	0.057	0	0	0.198	0.01
*Scomber japonicus*	0	0.057	0	0	0.198	0.25
*Semicossyphus pulcher*	0	0.057	0	0	0	0.01
*Seriphus politus*	0	0.029	0.050	0.1	0	0.02
*Trachurus symmetricus*	0	0	0	0	0.050	0.01
*Xenistius californiensis*	0	0	0	0.1	0.198	0.3
Total	100	175	101	100	101	100

Note

Total indicated is the total number of fish eggs used to construct the mock community.

In addition to the mock communities, we analyzed one large natural plankton sample form the kelp forest that contained approximately 4,500 fish eggs. From this large collection, two random samples of 500 eggs were processed separately using the metabarcoding protocol described above for the mock communities. Another subset of 1,000 fish eggs from the same collection was individually Sanger sequenced using methods described above (i.e., traditional barcoding).

Each mock community (6) and natural community (2) was amplified in four replicate PCRs with COI and 16S primer pairs separately (8 communities × 2 primer sets × 4 replicates = 64 libraries). Then, each of the six mock communities was again amplified in four reactions and replicate reactions were pooled before sequencing to compare the effects of pooling samples after amplification and before sequencing (6 mock communities × 2 primer sets = 12 libraries). Additionally, mock communities one through four were amplified in four replicate reactions with COI and 16S primer pairs in the same reaction to compare the effects of multiplexing primer pairs to single primer pair reactions (4 mock communities × 1 primer combination × 4 replicates = 16 libraries). Mock communities five and six were not included multiplexed with 16S and COI due to limitations on space in our sequencing run.

After amplification, PCR products were cleaned using Sephadex G‐50 Fine and quantified using Qubit 2.0 Fluorometer (ds HS Assay, Life Technologies). Samples were diluted to 0.02 ng/µl. A total of 92 libraries were then prepared using the Nextera XT Illumina protocol (Table S1). Dual‐indexed libraries were sequenced using Illumina MiSeq paired‐end sequencing with 2 × 300 cycles. The Nextera XT protocol fragments amplicons and may create potential bias by obtaining more than one tagged fragment from a given PCR amplicon. However, since the expected amplicon from each species is the same length, the probability of sequencing multiple fragments from a single amplicon is equal across species and should not lead to any systematic bias. We report data collected from all 92 libraries that were constructed and sequenced.

### 2.4 Data preprocessing and taxonomic assignment

Overall quality of MiSeq reads was evaluated in FastQC. The Nextera transposase sequences were trimmed from the target region. Low‐quality tails and Nextera adaptors were trimmed from the sequencing reads in CLC Genomics Workbench (Qiagen) with quality limit 0.01. Only reads with at least 100 retained base pairs were used in the analysis (details of the algorithm and its implementation are available in the CLC Genomics Workbench user manual). Broken pairs (paired‐end reads that were unmerged) and orphan reads were discarded. For each library, species were identified by mapping the reads to a reference library constructed from over 1,000 fish sequences of COI and 16S sequences (Supporting information). Two species (*Seriphus politus* and *Etrumeus acuminatus*) included in our constructed libraries did not have 16S rRNA sequences in NCBI. We obtained tissue samples from voucher specimens for these species from the Scripps Institution of Oceanography Marine Vertebrate Collection, extracted and sequenced their DNA and submitted these sequences to GenBank (accession numbers: MH714866 and MH718435). The total number of reads per species was mapped and counted using RNA‐seq analysis tools in CLC at 97% similarity to a local reference database. While this method may result in some loss of taxonomic signal via fragmentation of amplicons, we accounted for this by only counting reads that mapped uniquely to one species.

### 2.5 Data analysis

All data analyses and statistics were conducted in R. Total reads that mapped to a given species were counted and divided by the total number of reads mapped for the library to find the proportion of that species in the library. For samples where COI and 16S were multiplexed, the total species proportion was calculated based on the number of reads for each primer in a library to allow for separate analysis of both primer sets in multiplexed samples. The average number of missed species per library for each primer set was determined, and differences across primer sets and input species were assessed by two‐way ANOVA. We modeled detection of low‐frequency species (<~10% of a mock community) using a logistic regression with primer set and species as categorical variables. Differences between observed and expected proportional read counts for each primer set were assessed by ANCOVA with expected proportions as a covariate, and primers as a categorical variable.

## 3 RESULTS

The total number of reads generated was 15,197,856, and the average length of each read was 300 bp. After filtering and trimming, 1,297,257 were retained. Filtering reads after trimming of the Illumina transposase resulted in the greatest loss of mappable reads. Reads in both directions were mapped to the reference library. The average number of reads for each library was 13,853 ± 670 *SEM*, with a minimum of 2,357 and maximum of 32,543 (Figure S1).

### 3.1 Detection in mock communities

**Figure 1:**
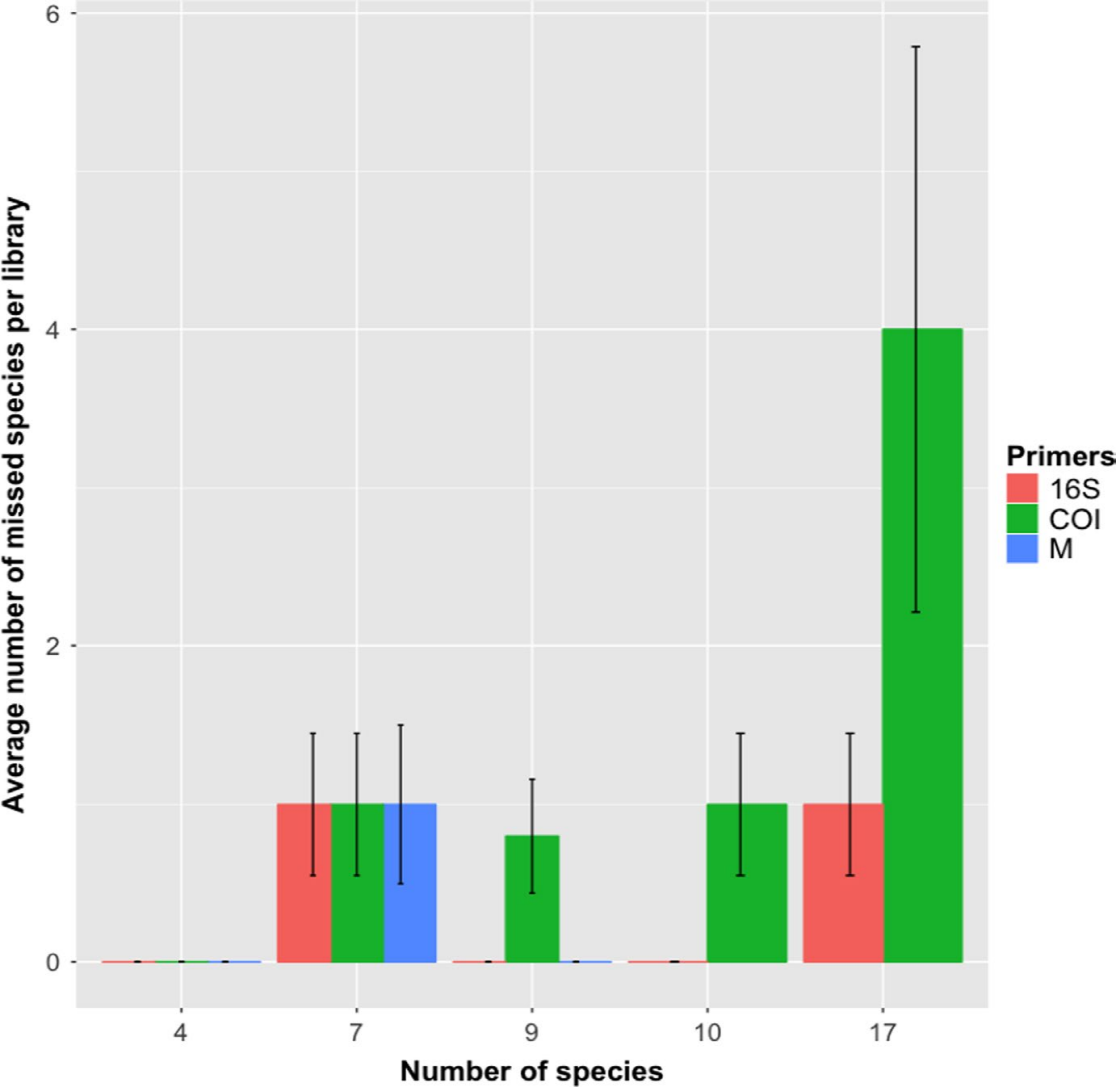
Average number of species that were not detected in libraries of mock communities of fish eggs with various numbers of input species from four to seventeen. Mock communities 5 and 6 were not analyzed with multiplexed primers

Overall, 60% of all libraries were able to detect all of the species that were input into mock communities (48/80). Forty percent (12/30) and sixty‐six percent (20/30) of all libraries amplified with COI primers or 16S primers were able to detect all the input species, respectively. These two primer sets were significantly different in their ability to detect all input species (*χ* ^2^ = 3.28, *df* = 1, *p* < .05). Seventy‐six percent of libraries that were multiplexed were able to detect all species; this was not significantly different from COI and 16S primer set's detection rate, though overall power is low (*χ* ^2^ = 1.36, *df* = 2, *p* > .05). Libraries that included more species were less likely to detect all of the species present (*F* = 163.50, 63.75, 37.94, *df* = 4,2,6, *p* < .01; Figure 1). However, the number of missed species depended on the primer set that was used in amplification with a significant interaction between primer set and the number of species in a mock community.

**Figure 2:**
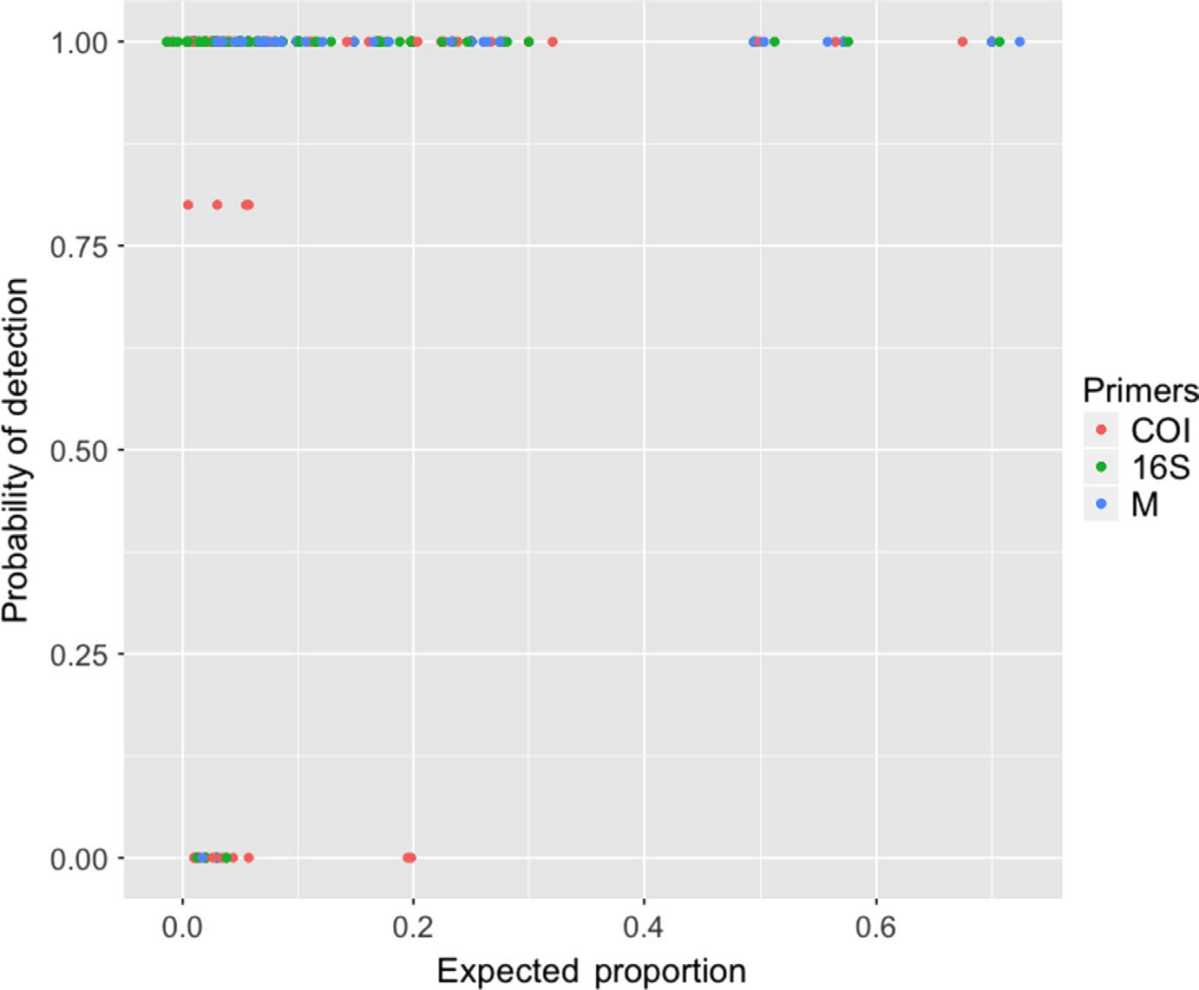
Probability of detection for a species was calculated based on the number of times that species was detected with specific primer sets at a certain expected proportion. Expected proportion based on the number of fish eggs of that species used in a mock community

Of the 23 different species included in the mock communities, 5 species were not consistently detected by COI primers: *Etrumeus acuminatus*, *Genyonemus lineatus*, *Pleuronichthys verticalis*, *Roncador stearnsii*, and *Sardinops sagax*. All of these species comprised <6% of the mock communities in which they were placed, with the exception of *Sardinops sagax*. With COI primers, *Sardinops sagax* was missed in three out of four libraries where it comprised 20% of the eggs. By comparison, the same mock communities amplified by 16S primers detected *Sardinops sagax* 100% of the time. This species in particular is known to not amplify well with COI primers (A. Harada, D. Kacev, pers comm) but can be easily identified morphologically (Harada et al., 2015). With the exception of *Sardinops sagax* amplified with COI primers, every species that was in a mock community at a frequency of 6% or greater was detected 100% of the time (Figure 2). Species that comprised <10% of mock community were detected at varying frequency depending on the species and the primer set that was used in amplification (*χ* ^2^ = 259.66, 11.78, *df* = 2, 20, *p* < .01; Figure S2). Of all the species included in the mock communities, only *Genyonemus lineatus* was missed by libraries amplified with all three primer combinations (COI, 16S, or multiplexed). This species was not detected in two of the mock communities (3,6) where it comprised 2%, and 3% of the eggs, respectively. It is remarkable that many species that were in mock communities at frequencies as low as 1% were reliably detected by both COI and 16S primer sets (Figure S2).

We detected low levels of false positives in our samples which were species that were not included in our mock community but found in the sequenced libraries. Ninety‐two percent of false positives that were detected comprised <1% of the total reads in the library (Figure S3). The median observed proportion of a species with a false positive was very low (0.04% of the reads). These reads were identified as thirty‐eight species, some of which were apparently low levels of cross‐contamination between libraries or mapping errors. It is also possible that some contamination is due to eDNA stuck on the fish egg surfaces (Fritts et al., 2019 and see discussion below; Table S1).

### 3.2 Relative proportions

**Figure 3:**
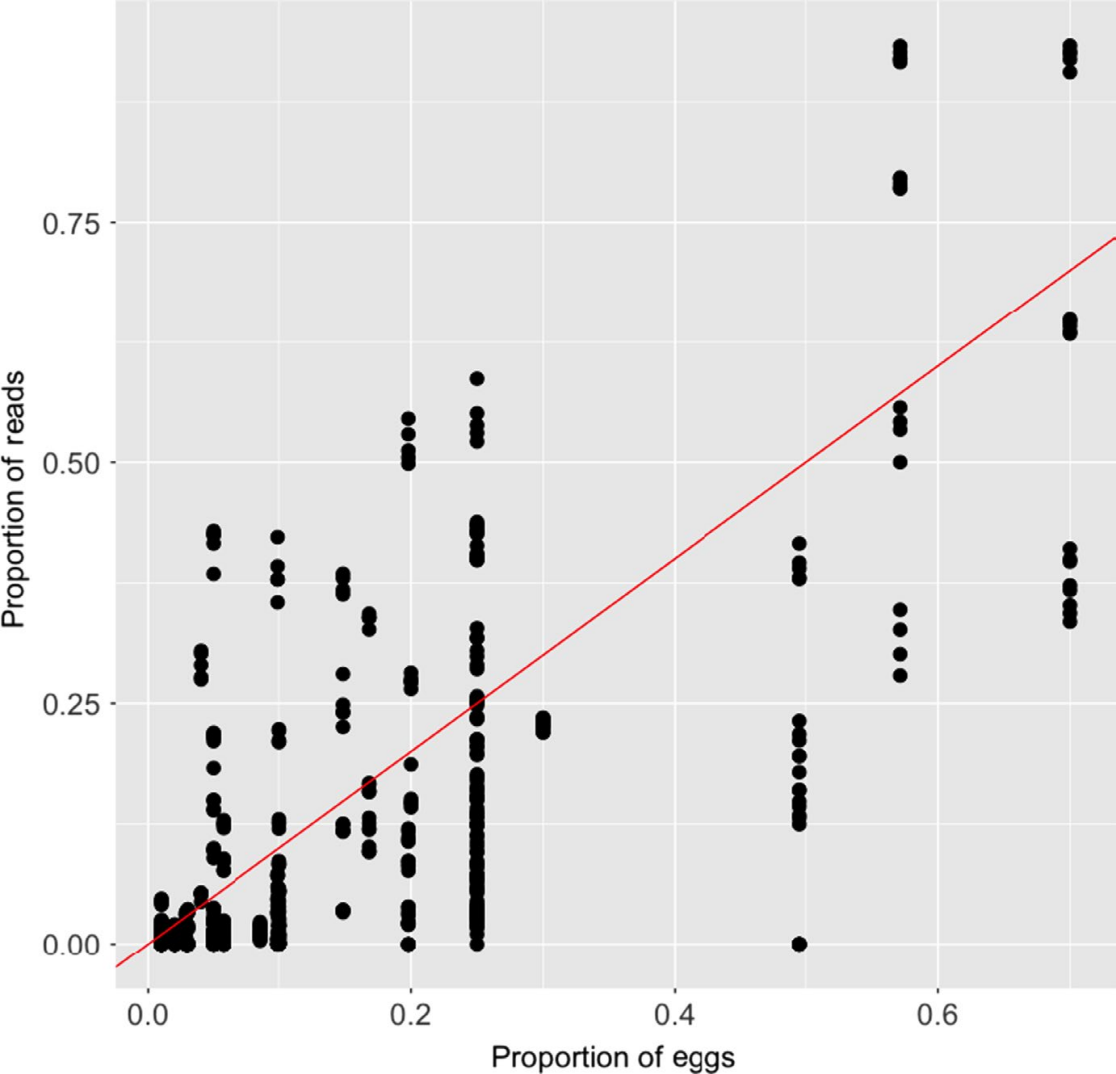
Linear regression between the proportions of eggs of a given species in relation to the number of reads detected of that species for all libraries. Regression line shown is 1:1 line. *R* ^2^ = .63 0.66 calculated around a 1:1 line

**Figure 4:**
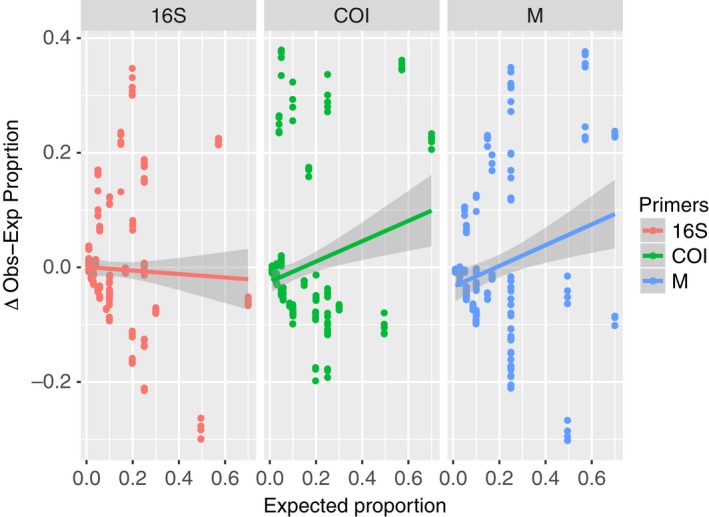
Analysis of covariance between the expected proportion of each species and the difference between the observed and expected with primers as a fixed factor. No significant differences were detected between primers. M indicated samples were multiplexed

**Figure 5:**
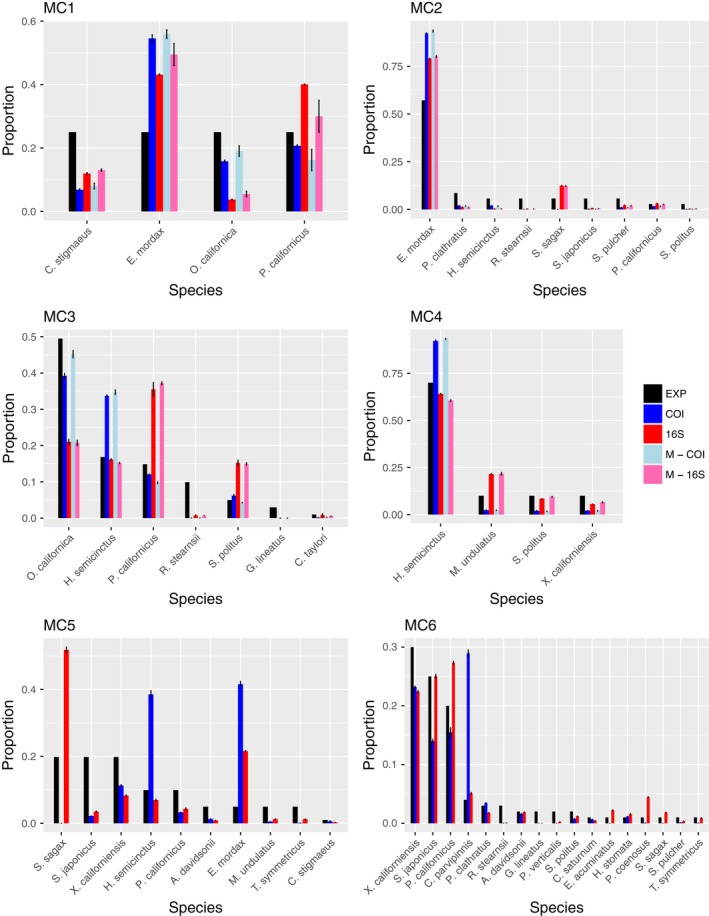
Summary of 4 replicate libraries for each mock community shown for each primer combinations tested. Expected proportion of species based on number of eggs shown in black. M indicates samples were multiplexed

**Figure 6:**
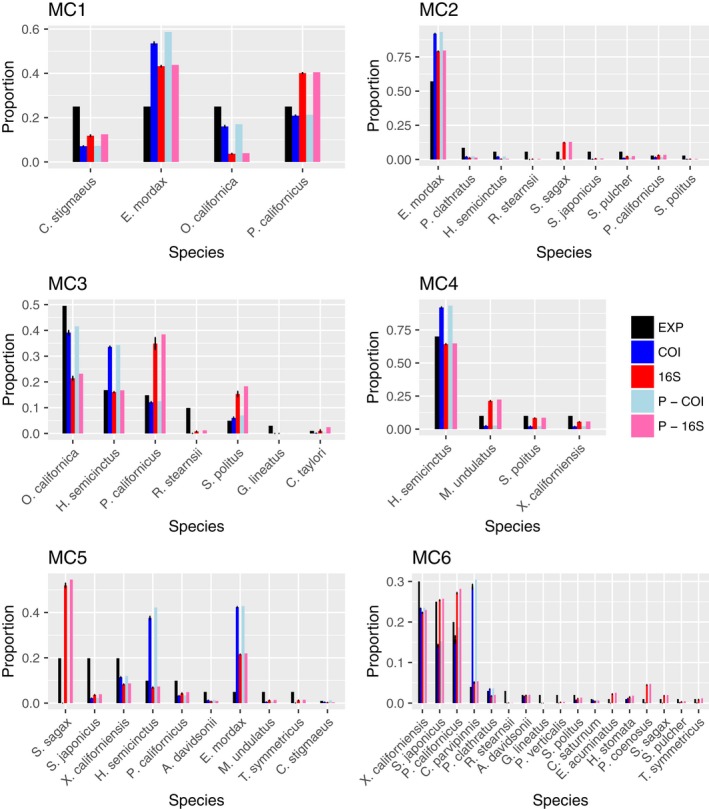
Summary of replicate libraries for each mock community shown for both primer sets, both pooled before sequencing and not pooled. Expected proportion of species based on number of eggs is shown in black. P indicates replicate PCRs were pooled before sequencing. Standard error is shown for replicate PCRs that were not pooled before sequencing

Across all libraries, we found a positive relationship between the expected and observed proportions of reads for each species in a library, (*R* ^2^ = .66) calculated around a 1:1 line (Figure 3). The absolute value of the differences between the observed and expected proportions ranged from 0.38 to 0. In the most extreme case, *E. mordax* comprised 5% of the eggs in mock community five (MC5) but accounted for 43% of the total reads in that library. Conversely, in some cases we found a <0.01% difference in proportion between the observed read count and expected number of reads based on number of eggs. While this is a large range of error, 74% of the differences between observed and expected proportions fell within 0 and 0.10 (Figure S4). The average and median difference in proportion were 0.084 and 0.053, respectively. Moreover, the relationship between the expected proportion and the difference between observed and expected differed depending on the primer set that was used, though the strength of that relationship is weak (Figure 4; *F* = 14.70, 0.076, 5.75, *p* < .01). There was a significant interaction between expected proportion and primers used. We observed that samples that were multiplexed or independently amplified with each primer set gave similar final estimates for each species (Figure 5; MC 1–4). We also observed that pooling samples before sequencing or combining replicate libraries after sequencing yielded extremely similar final estimates of proportional abundance for each species (Figure 6).

### 3.3 Analysis of a natural collection

**Figure 7:**
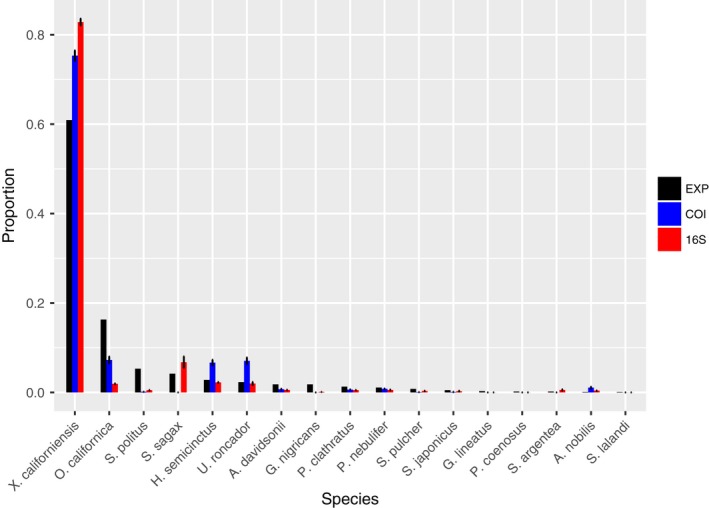
Summary of 4 replicate libraries for unknown natural community shown for each primer combination tested. Expected proportion of species based on number of eggs that were Sanger sequenced from the same collection is shown in black

A large natural sample of over 4,500 eggs was obtained from the kelp forest sampling site on 17 August 2017. This sample permitted side‐by‐side comparison of traditional barcoding to metabarcoding. For barcoding, 1,000 individual eggs were sequenced using our standard Sanger sequencing approach to determine species present and their relative abundance in the sample (Table 2). Two samples containing 500 fish eggs each were randomly selected from the same natural collection and analyzed by our metabarcoding protocol. Unlike the mock communities with known composition, here the standard for comparison is the set of Sanger sequenced eggs from the same sample. From Sanger sequencing of 1,000 fish eggs, we found a total of 17 species. Of the sixteen libraries, eight libraries amplified with 16S primers contained more than 17 species and the other eight libraries amplified with COI primers contained <17 species. Figure 7 shows the species and the relative proportion they were found in the libraries for both primer sets used (eight libraries for each primer). Both COI and 16S primers found all species that comprised over three percent of the subset eggs that we Sager sequenced. Species below 3% were either detected by one primer set or neither (Table 2). It is important to note that the absence of some low‐abundance species from the metabarcoded libraries could simply reflect random sampling error (we expect some of the rarer species to be missing in a given population sample).

**Table 2:** List of species observed in a field collection used to compare Sanger sequencing with metabarcoding

Species	Proportion	16S‐UK1	16S‐UK2	COI‐UK1	COI‐UK2
*Xenistius californiensis*	0.609	0.84695	0.81070	0.77886	0.72813
*Oxyjulis californica*	0.163	0.01808	0.02054	0.05274	0.09221
*Seriphus politus*	0.053	0.00589	0.00387	0.00161	0.00156
*Sardinops sagax*	0.042	0.03390	0.10006	0.00000	0.00001
*Halichoeres semicinctus*	0.028	0.02034	0.02495	0.05101	0.08306
*Umbrina roncador*	0.023	0.02868	0.01108	0.08828	0.05274
*Anisotremus davidsonii*	0.018	0.00485	0.00652	0.00419	0.01023
*Girella nigricans*	0.018	0.00131	0.00105	0.00006	0.00008
*Paralabrax clathratus*	0.013	0.00609	0.00406	0.00615	0.00663
*Paralabrax nebulifer*	0.011	0.00746	0.00389	0.00852	0.00767
*Semicossyphus pulcher*	0.008	0.00482	0.00181	0.00075	0.00090
*Scomber japonicus*	0.005	0.00502	0.00067	0.00174	0.00041
*Genyonemus lineatus*	0.003	0.00000	0.00000	0.00000	0.00000
*Pleuronichthys coenosus*	0.002	0.00000	0.00002	0.00000	0.00000
*Sphyraena argentea*	0.002	0.00876	0.00272	0.00002	0.00001
*Atractoscion nobilis*	0.001	0.00323	0.00513	0.00581	0.01584
*Seriola lalandi*	0.001	0.00000	0.00000	0.00000	0.00000
*Citharichthys stigmaeus*	NA	0.00060	0.00079	0.00002	0.00047
*Cynoscion parvipinnis*	NA	0.00007	0.00029	NA	NA
*Engraulis mordax*	NA	0.00022	0.00005	0.00001	0.00002
*Haemulon flaviguttatum*	NA	0.00015	0.00005	NA	NA
*Hermosilla azurea*	NA	0.00237	0.00167	NA	NA
*Menticirrhus undulatus*	NA	0.00119	NA	0.00021	NA
*Paralabrax maculatofasciatus*	NA	0.00002	NA	NA	0.00001
*Paralichthys californicus*	NA	NA	0.00002	0.00001	NA
*Pleuronichthys ritteri*	NA	NA	0.00002	NA	NA
*Roncador stearnsii*	NA	NA	0.00002	NA	NA
*Sardinops sagax*	NA	NA	NA	NA	0.00002

Note

Results of Sanger sequencing of 1,000 individual eggs form this collection and subsequently compared to the two samples, UK1 and UK2, of 500 eggs each from the same collection that were sequenced using metabarcoding protocols. The pools of 500 eggs were amplified with 16S and COI primers and sequenced using Illumina MiSeq.

## 4 DISCUSSION

Our overall objective was to evaluate the efficacy of metabarcoding protocols on ichthyoplankton to determine species presence and relative abundance using mock communities of fish eggs. These communities were constructed using both even and skewed species abundances. We also used a natural collection to compare results from metabarcoding and traditional DNA barcoding methods. Our results demonstrate that metabarcoding protocols were able to (a) detect all high‐abundance species and many of the low‐abundance species placed mock communities, and (b) determine high‐abundance species and low‐abundance species with both even and skewed species abundances.

### 4.1 Detection

In general, species that comprised over six percent of a mock community were reliably detected in our metabarcoding results. Species that comprised <6% of a mock community were detected with varying frequency depending on the primers used and its relative proportion in the mock community. As the number of species per library increased, the ability to detect all species decreased. Furthermore, for both for COI and 16S libraries, the highest variability in number of species detected occurs in libraries with the most species (Figure 1), which is likely due to stochastic effects of PCR as well as decreasing the relative proportion of each species (Deagle, Jarman, Coissac, Pompanon, & Taberlet, 2014; Pawluczyk et al., 2015). These results imply that as the number of species in a sample increases, there would be an increased likelihood that sequencing may miss a given species.

Detection of low‐abundance species varied with the primer set that was used in amplification. This variation is likely the result of differences in the binding efficiencies of primer pairs across template differences for certain species (Clarke, Soubrier, Weyrich, & Cooper, 2014; Deagle et al., 2014), given primer pairs may not work well with certain species and therefore decrease ability to detect that species in a library. For example, although *Sardinops sagax* eggs comprised 20% of a mock community, it was not detected in our libraries constructed with COI primers. This is consistent with other observations that these primers do not work well with this particular species (in fact, *S. sagax* eggs are morphologically distinct so they have been omitted from some molecular identification studies (e.g., Harada et al., 2015)). Similarly, others have found that “universal” primers did not amplify groups of taxa so specific primers had to be designed to detect these species in eDNA studies (Kelly et al., 2014; Miya et al., 2015). Because the degree of amplification bias depends on the mix of species in the constructed mock community, we cannot generalize how this bias affects species detection thresholds. Full mitochondrial genome sequences for these fish species would be necessary in order to evaluate the effects of primer‐template mismatches on amplification bias in the constructed libraries. Currently, there are relatively few full mitochondrial genome sequences for fish species published in available databases; however, as they become available, future studies could tease apart the effects of amplification bias from other variables that may impact detection thresholds.

We found generalized differences between primer pairs and their ability to detect all species in a mock community, with 16S detecting proportionally more species than COI in our study. Similarly, Evans et al. (2016) found that only two of six primer sets used in an experimental eDNA study were able to recover the complete species assemblages. These results highlight the importance of using multiple primer pairs to improve estimates of species presence in a given sample. Recent publications have used 12S ribosomal markers for identification, but due to lack of barcode database in our region, we were unable to use this marker (Evans et al., 2016; Miya et al., 2015). Currently, there is an effort to develop a barcode database for this marker by NOAA Southwest Fisheries Science Center and it could be included along with COI and 16S in future studies (D. Kacev pers. comm). Our results suggest that in moderately diverse species assemblages, rare species can be detected using a combination of markers. For example, we have found a maximum number of 19 species in a single collection and total number of 49 species observed from sequencing over 25,000 eggs collected from the Scripps Pier weekly over a period of 6 years (Duke et al., 2018; Harada et al., 2015). In contrast, Ahern et al. (2018) identified over 150 species (and up to 38 species in a single collection) from the subtropical southeastern Gulf of California. The efficacy of metabarcoding in such diverse fish communities remains to be explored.

### 4.2 Relative proportion

There was a positive relationship between the proportions of reads obtained from a given species and the proportion of eggs that was combined in the mock community, though there was reasonable amount of variability (*R* ^2^ = .66). These results are consistent with other metabarcoding studies that have found a positive relationship between species abundance and species read abundance (Evans et al., 2016; Thomsen et al., 2012). While most of the differences between observed and expected proportions fell between 0 and 0.1, in some cases it was as high as 0.38 (Figure S4). This large variability could result from PCR amplification bias or interspecific differences in the amount of mtDNA per egg (see below). Overrepresentation or underrepresentation based on the primers may result from differences in primer binding efficiencies across species (Deagle et al., 2014). For example, some species were detected at very low proportions (<0.01), while other remained undetected at the same input proportion. A similar study found that species could be detected at biomass percentages as low as 0.02%, but detection limits varied among species and could vary considerably from expected biomass ratios (Evans et al., 2016; Hatzenbuhler et al., 2017).

When a low‐abundance species is undetected or underrepresented (i.e., failed amplification), this will result in proportional overrepresentation of other taxa in the sample (Deagle et al., 2014). There were species that were consistently overrepresented in our samples across primer sets and across mock communities. For example, the northern anchovy (*Engraulis mordax*) in MC1, MC2, and MC5 is overrepresented across all primer sets used (Figure 5). This implies that in addition to primer binding efficacies, there may be inherent species‐specific differences driving this pattern. This could arise from differences in mitochondrial copy number or DNA extraction efficiencies across species (Schloss et al., 2011). Although we did not measure egg size or developmental stage of fish eggs, a similar study that measured biomass of larval fish of similar developmental stages found that metabarcoding results were skewed from expected biomass percentages (Hatzenbuhler et al., 2017). Mitochondrial density and mtDNA copy number in larval fishes are not well understood and may affect detection and proportional species estimates in diverse samples. Our results highlight the need for additional research on the effects of mitochondrial variation among fish embryos and its influence on biodiversity estimates for metabarcoding data.

Quite interestingly, our results showed that regardless if primer pairs were used independently or multiplexed, estimates from each amplicon (COI or 16S) were consistent across species (Figure 5; MC 1–4). Either multiplexing or amplifying primers in separate reactions yielded similar final estimates of species proportions. Similarly, our comparisons between replicates that were pooled before sequencing or after sequencing showed that both methods resulted in very similar estimates of species proportion though notably also suffered from similar amplification biases.

We detected low levels of false positives in our samples, which are likely due to mapping error, cross‐contamination in the laboratory, or potentially eDNA stuck on the fish egg surfaces (Fritts et al., 2019). Mapping error was particularly easy to identify, because some sequences mapped to species that were not included in any of the constructed libraries. These were species that we have not previously seen in our laboratory but are related to species we have found at Scripps Pier (Duke et al., 2018). For example, several 16S reads were attributed to *Seriola rivoliana* (Table S1), a species we have not observed in our 6 years of sampling. However, we have found *Seriola lalandi* within the same genus and a comparison of 16S rRNA sequences on GenBank shows that the species divergence is approximately 3%, right at our mapping cutoff. There were also species that we have seen in our laboratory that likely resulted from low‐level cross‐contamination of samples during PCR cleanup or library prep. These observations suggest that the move to next‐gen sequencing requires additional care be given to laboratory procedures needed to prevent low levels of post‐PCR cross‐contamination that generally have little impact on Sanger sequencing results. Notably, most false positives comprised a very small proportion of the overall library and could be eliminated by removing reads that fall below some threshold proportion of the total library. Additionally, future studies should include negative controls, which are often uncommon in metabarcoding studies, but could help reveal sources of laboratory contamination. Our mock communities present a kind of negative control in that the eggs in each library were known entities based on standard barcoding; for example, MC1 and MC4 were “negative controls” for all species other than 4 included in each mix. However, true negative controls in addition to mock communities may help to understand rates of false positives in future sequencing analyses.

Considering the broad range of factors that could reduce the accuracy of metabarcoding, we found our results rather promising. First, we note that are many different ways to prepare samples (we used bead beating and Qiagen's DNeasy Blood and Tissue Kit), amplify target genes (we used a nonproofreading GoTaq), prepare sequencing libraries (we used Illumina's Nextera XT kit); we did not systematically explore other options in this study. Although GoTaq does not have 3′→5′ exonuclease activity (“proofreading”), PCR error is not likely to be a confounding issue when sequence matching for species identification is only at 97% stringency; in fact, PCR error with Taq is likely one or more orders of magnitude lower than Illumina sequencing error (see Pfeiffer et al., 2018). Second, variation in cell number, mitochondrial density, and mtDNA copy number among developmental stages within species and between species could lead to biases; these issues will likely have greatest impacts when few eggs are pooled into a single sample. Third, PCR amplification biases add to inaccuracies in analyses of mixed‐species samples (Clarke et al., 2014; Polz & Cavanaugh, 1998). Rare species may go undetected using universal primers, but when desired, they may be recovered using species or group‐specific primers (though this may interfere with relative proportion estimates). Despite these issues, we show that our techniques use can give information on both abundant and less abundant species; however, obtaining absolute species proportions as estimates of community composition from metabarcoding remains challenging. If metabarcoding is to be employed for monitoring and management purposes, continued ground‐truthing using multiple markers should be done to document primer biases for species of interest in order to be able to make appropriate inferences about species presence and relative proportion.

## CONFLICT OF INTEREST

None declared.

## AUTHOR CONTRIBUTIONS

Elena Duke performed research, analyzed data, and wrote the paper. Ron Burton designed research, wrote the paper, and is the principal investigator.

## Supporting information

Supinfo

## Data Availability

Data are available at Dryad Digital Repository at: https://doi.org/10.5061/dryad.zw3r22854. Two sequences used in our reference library are on GenBank (accession numbers MH714866 and MH718435).
