# Supplementary material for: Efficacy of metabarcoding for identification of fish eggs evaluated with mock communities
Source: Ecol Evol. 2020 Mar 3;10(7):3463–76. doi: 10.1002/ece3.6144 (PMC7141059; doi:10.1002/ece3.6144)
Supplement: Supplementary file 1 — Supinfo [file ECE3-10-3463-s001.docx]

Supplementary Materials

Figure S1. Box plot of total reads in each library (n = 92).

Figure S2. Proportion of species detected was calculated based on the number of times that species was detected with specific primer sets at a certain expected proportion (less than 10%). Expected proportion based on the number of fish eggs of that species used in a mock community.

Same data as Figure 2, listing only species that comprised 10% or less of a mock community.


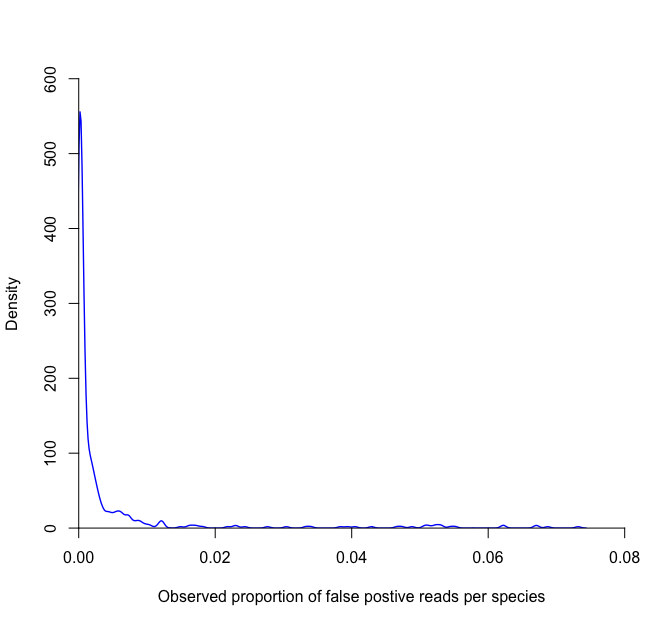


Figure S3. Density plot of false positives observed in constructed libraries.


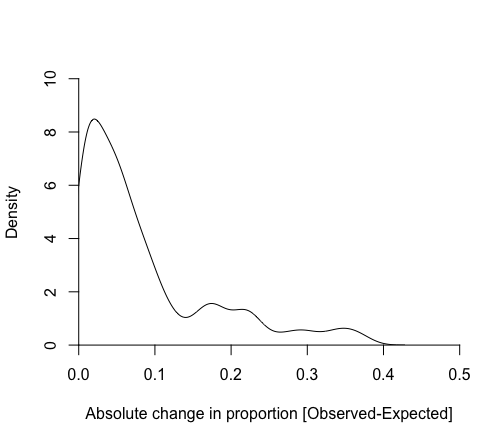


Figure S4. Density plot of the absolute value of the difference between observed and expected proportion in all libraries for all species. Observed and expected proportions were based on read count and number of eggs in the mock community a given species, respectively.

Table S1. List of all species that were identified as a false positive in any of the mock communities constructed across all markers with observed read count and proportion of total read count shown.

| **Library** | **MC** | **Primers** | **Species** | **Locus** | **Observed read count** | **Observed Proportion** |
| --- | --- | --- | --- | --- | --- | --- |
| E1 | MC1 | COI | Halichoeres semicinctus | COI | 21 | 0.00497 |
| E1 | MC1 | COI | Semicossyphus pulcher | COI | 5 | 0.00118 |
| E1 | MC1 | COI | Paralabrax clathratus | COI | 3 | 0.00071 |
| E1 | MC1 | COI | Cynoscion parvipinnis | COI | 2 | 0.00047 |
| E1 | MC1 | COI | Scomber japonicus | COI | 1 | 0.00024 |
| F1 | MC2 | COI | Citharichthys stigmaeus | COI | 10 | 0.00226 |
| F1 | MC2 | COI | Citharichthys sordidus | COI | 1 | 0.00023 |
| G1 | MC3 | COI | Engraulis mordax | COI | 110 | 0.04663 |
| G1 | MC3 | COI | Citharichthys stigmaeus | COI | 41 | 0.01738 |
| G1 | MC3 | COI | Xenistius californiensis | COI | 6 | 0.00254 |
| G1 | MC3 | COI | Semicossyphus pulcher | COI | 3 | 0.00127 |
| G1 | MC3 | COI | Umbrina roncador | COI | 3 | 0.00127 |
| G1 | MC3 | COI | Pleuronichthys ritteri | COI | 2 | 0.00085 |
| G1 | MC3 | COI | Cheilotrema saturnum | COI | 1 | 0.00042 |
| H1 | MC4 | COI | Citharichthys stigmaeus | COI | 31 | 0.00457 |
| H1 | MC4 | COI | Paralichthys californicus | COI | 8 | 0.00118 |
| H1 | MC4 | COI | Citharichthys sordidus | COI | 3 | 0.00044 |
| H1 | MC4 | COI | Etrumeus teres | COI | 3 | 0.00044 |
| H1 | MC4 | COI | Oxyjulis californica | COI | 2 | 0.00030 |
| H1 | MC4 | COI | Cheilotrema saturnum | COI | 1 | 0.00015 |
| H1 | MC4 | COI | Paralabrax clathratus | COI | 1 | 0.00015 |
| A2 | MC5 | COI | Umbrina roncador | COI | 11 | 0.00070 |
| A2 | MC5 | COI | Cheilotrema saturnum | COI | 3 | 0.00019 |
| A2 | MC5 | COI | Etrumeus teres | COI | 3 | 0.00019 |
| A2 | MC5 | COI | Oxyjulis californica | COI | 3 | 0.00019 |
| A2 | MC5 | COI | Sardinops sagax | COI | 2 | 0.00013 |
| A2 | MC5 | COI | Citharichthys sordidus | COI | 1 | 0.00006 |
| B2 | MC6 | COI | Etrumeus teres | COI | 943 | 0.05237 |
| B2 | MC6 | COI | Halichoeres semicinctus | COI | 709 | 0.03937 |
| B2 | MC6 | COI | Citharichthys stigmaeus | COI | 289 | 0.01605 |
| B2 | MC6 | COI | Umbrina roncador | COI | 123 | 0.00683 |
| B2 | MC6 | COI | Citharichthys sordidus | COI | 29 | 0.00161 |
| B2 | MC6 | COI | Engraulis mordax | COI | 7 | 0.00039 |
| B2 | MC6 | COI | Oxyjulis californica | COI | 5 | 0.00028 |
| B2 | MC6 | COI | Menticirrhus undulatus | COI | 2 | 0.00011 |
| C2 | MC1 | 16S | Sardinops sagax | 16S | 79 | 0.00556 |
| C2 | MC1 | 16S | Semicossyphus pulcher | 16S | 46 | 0.00324 |
| C2 | MC1 | 16S | Scomber japonicus | 16S | 38 | 0.00267 |
| C2 | MC1 | 16S | Halichoeres semicinctus | 16S | 2 | 0.00014 |
| D2 | MC2 | 16S | Anchoa delicatissima | 16S | 4 | 0.00022 |
| D2 | MC2 | 16S | Oxyjulis californica | 16S | 3 | 0.00017 |
| D2 | MC2 | 16S | Citharichthys stigmaeus | 16S | 1 | 0.00006 |
| E2 | MC3 | 16S | Engraulis mordax | 16S | 708 | 0.06694 |
| E2 | MC3 | 16S | Pleuronichthys ritteri | 16S | 94 | 0.00889 |
| E2 | MC3 | 16S | Semicossyphus pulcher | 16S | 75 | 0.00709 |
| E2 | MC3 | 16S | Citharichthys stigmaeus | 16S | 48 | 0.00454 |
| E2 | MC3 | 16S | Girella nigricans | 16S | 32 | 0.00303 |
| E2 | MC3 | 16S | Scomber japonicus | 16S | 21 | 0.00199 |
| E2 | MC3 | 16S | Menticirrhus undulatus | 16S | 20 | 0.00189 |
| E2 | MC3 | 16S | Xenistius californiensis | 16S | 9 | 0.00085 |
| E2 | MC3 | 16S | Pleuronichthys coenosus | 16S | 4 | 0.00038 |
| E2 | MC3 | 16S | Sardinops sagax | 16S | 4 | 0.00038 |
| E2 | MC3 | 16S | Cheilotrema saturnum | 16S | 1 | 0.00009 |
| E2 | MC3 | 16S | Etrumeus acuminatus | 16S | 1 | 0.00009 |
| E2 | MC3 | 16S | Paralabrax maculatofasciatus | 16S | 1 | 0.00009 |
| F2 | MC4 | 16S | Oxyjulis californica | 16S | 25 | 0.00242 |
| F2 | MC4 | 16S | Etrumeus acuminatus | 16S | 2 | 0.00019 |
| G2 | MC5 | 16S | Anchoa delicatissima | 16S | 2 | 0.00014 |
| G2 | MC5 | 16S | Haemulon flaviguttatum | 16S | 1 | 0.00007 |
| G2 | MC5 | 16S | Hermosilla azurea | 16S | 1 | 0.00007 |
| H2 | MC6 | 16S | Citharichthys stigmaeus | 16S | 368 | 0.02181 |
| H2 | MC6 | 16S | Halichoeres semicinctus | 16S | 61 | 0.00362 |
| H2 | MC6 | 16S | Seriola lalandi | 16S | 24 | 0.00142 |
| H2 | MC6 | 16S | Seriola rivoliana | 16S | 11 | 0.00065 |
| H2 | MC6 | 16S | Naucrates ductor | 16S | 9 | 0.00053 |
| H2 | MC6 | 16S | Citharichthys xanthostigma | 16S | 5 | 0.00030 |
| H2 | MC6 | 16S | Citharichthys sordidus | 16S | 2 | 0.00012 |
| H2 | MC6 | 16S | Engraulis mordax | 16S | 2 | 0.00012 |
| H2 | MC6 | 16S | Haemulon flaviguttatum | 16S | 2 | 0.00012 |
| H2 | MC6 | 16S | Chilara taylori | 16S | 1 | 0.00006 |
| H2 | MC6 | 16S | Paralabrax maculatofasciatus | 16S | 1 | 0.00006 |
| A3 | MC1 | COI | Halichoeres semicinctus | COI | 133 | 0.00833 |
| A3 | MC1 | COI | Semicossyphus pulcher | COI | 30 | 0.00188 |
| A3 | MC1 | COI | Cynoscion parvipinnis | COI | 13 | 0.00081 |
| A3 | MC1 | COI | Scomber japonicus | COI | 10 | 0.00063 |
| A3 | MC1 | COI | Citharichthys sordidus | COI | 5 | 0.00031 |
| A3 | MC1 | COI | Paralabrax clathratus | COI | 3 | 0.00019 |
| A3 | MC1 | COI | Seriphus politus | COI | 3 | 0.00019 |
| A3 | MC1 | COI | Etrumeus teres | COI | 1 | 0.00006 |
| B3 | MC1 | COI | Halichoeres semicinctus | COI | 129 | 0.00843 |
| B3 | MC1 | COI | Semicossyphus pulcher | COI | 12 | 0.00078 |
| B3 | MC1 | COI | Scomber japonicus | COI | 10 | 0.00065 |
| B3 | MC1 | COI | Citharichthys sordidus | COI | 6 | 0.00039 |
| B3 | MC1 | COI | Paralabrax clathratus | COI | 5 | 0.00033 |
| B3 | MC1 | COI | Cynoscion parvipinnis | COI | 2 | 0.00013 |
| B3 | MC1 | COI | Seriphus politus | COI | 1 | 0.00007 |
| B3 | MC1 | COI | Xenistius californiensis | COI | 1 | 0.00007 |
| C3 | MC1 | COI | Halichoeres semicinctus | COI | 116 | 0.01026 |
| C3 | MC1 | COI | Semicossyphus pulcher | COI | 15 | 0.00133 |
| C3 | MC1 | COI | Scomber japonicus | COI | 9 | 0.00080 |
| C3 | MC1 | COI | Cynoscion parvipinnis | COI | 8 | 0.00071 |
| C3 | MC1 | COI | Paralabrax clathratus | COI | 4 | 0.00035 |
| C3 | MC1 | COI | Citharichthys sordidus | COI | 1 | 0.00009 |
| C3 | MC1 | COI | Seriphus politus | COI | 1 | 0.00009 |
| D3 | MC1 | COI | Halichoeres semicinctus | COI | 178 | 0.00961 |
| D3 | MC1 | COI | Semicossyphus pulcher | COI | 27 | 0.00146 |
| D3 | MC1 | COI | Cynoscion parvipinnis | COI | 11 | 0.00059 |
| D3 | MC1 | COI | Paralabrax clathratus | COI | 11 | 0.00059 |
| D3 | MC1 | COI | Scomber japonicus | COI | 11 | 0.00059 |
| D3 | MC1 | COI | Seriphus politus | COI | 5 | 0.00027 |
| D3 | MC1 | COI | Citharichthys sordidus | COI | 4 | 0.00022 |
| D3 | MC1 | COI | Etrumeus teres | COI | 2 | 0.00011 |
| D3 | MC1 | COI | Xenistius californiensis | COI | 1 | 0.00005 |
| E3 | MC2 | COI | Citharichthys stigmaeus | COI | 2 | 0.00011 |
| E3 | MC2 | COI | Xenistius californiensis | COI | 2 | 0.00011 |
| E3 | MC2 | COI | Menticirrhus undulatus | COI | 1 | 0.00005 |
| E3 | MC2 | COI | Oxyjulis californica | COI | 1 | 0.00005 |
| E3 | MC2 | COI | Umbrina roncador | COI | 1 | 0.00005 |
| F3 | MC2 | COI | Citharichthys stigmaeus | COI | 2 | 0.00017 |
| F3 | MC2 | COI | Xenistius californiensis | COI | 1 | 0.00008 |
| G3 | MC2 | COI | Citharichthys stigmaeus | COI | 1 | 0.00009 |
| H3 | MC2 | COI | Xenistius californiensis | COI | 4 | 0.00022 |
| H3 | MC2 | COI | Citharichthys stigmaeus | COI | 3 | 0.00017 |
| H3 | MC2 | COI | Oxyjulis californica | COI | 1 | 0.00006 |
| H3 | MC2 | COI | Umbrina roncador | COI | 1 | 0.00006 |
| A4 | MC3 | COI | Engraulis mordax | COI | 587 | 0.05146 |
| A4 | MC3 | COI | Citharichthys stigmaeus | COI | 55 | 0.00482 |
| A4 | MC3 | COI | Xenistius californiensis | COI | 25 | 0.00219 |
| A4 | MC3 | COI | Semicossyphus pulcher | COI | 20 | 0.00175 |
| A4 | MC3 | COI | Scomber japonicus | COI | 3 | 0.00026 |
| A4 | MC3 | COI | Girella nigricans | COI | 2 | 0.00018 |
| A4 | MC3 | COI | Etrumeus teres | COI | 1 | 0.00009 |
| A4 | MC3 | COI | Umbrina roncador | COI | 1 | 0.00009 |
| B4 | MC3 | COI | Engraulis mordax | COI | 637 | 0.04883 |
| B4 | MC3 | COI | Citharichthys stigmaeus | COI | 78 | 0.00598 |
| B4 | MC3 | COI | Semicossyphus pulcher | COI | 36 | 0.00276 |
| B4 | MC3 | COI | Xenistius californiensis | COI | 35 | 0.00268 |
| B4 | MC3 | COI | Etrumeus teres | COI | 3 | 0.00023 |
| B4 | MC3 | COI | Scomber japonicus | COI | 3 | 0.00023 |
| C4 | MC3 | COI | Engraulis mordax | COI | 834 | 0.04737 |
| C4 | MC3 | COI | Citharichthys stigmaeus | COI | 124 | 0.00704 |
| C4 | MC3 | COI | Semicossyphus pulcher | COI | 33 | 0.00187 |
| C4 | MC3 | COI | Xenistius californiensis | COI | 23 | 0.00131 |
| C4 | MC3 | COI | Scomber japonicus | COI | 7 | 0.00040 |
| C4 | MC3 | COI | Umbrina roncador | COI | 4 | 0.00023 |
| C4 | MC3 | COI | Girella nigricans | COI | 3 | 0.00017 |
| C4 | MC3 | COI | Pleuronichthys ritteri | COI | 2 | 0.00011 |
| C4 | MC3 | COI | Menticirrhus undulatus | COI | 1 | 0.00006 |
| D4 | MC3 | COI | Engraulis mordax | COI | 955 | 0.05446 |
| D4 | MC3 | COI | Citharichthys stigmaeus | COI | 98 | 0.00559 |
| D4 | MC3 | COI | Xenistius californiensis | COI | 50 | 0.00285 |
| D4 | MC3 | COI | Semicossyphus pulcher | COI | 39 | 0.00222 |
| D4 | MC3 | COI | Paralabrax clathratus | COI | 8 | 0.00046 |
| D4 | MC3 | COI | Scomber japonicus | COI | 5 | 0.00029 |
| D4 | MC3 | COI | Citharichthys sordidus | COI | 4 | 0.00023 |
| D4 | MC3 | COI | Cynoscion parvipinnis | COI | 4 | 0.00023 |
| D4 | MC3 | COI | Umbrina roncador | COI | 2 | 0.00011 |
| D4 | MC3 | COI | Girella nigricans | COI | 1 | 0.00006 |
| D4 | MC3 | COI | Pleuronichthys ritteri | COI | 1 | 0.00006 |
| E4 | MC4 | COI | Citharichthys stigmaeus | COI | 44 | 0.00290 |
| E4 | MC4 | COI | Etrumeus teres | COI | 3 | 0.00020 |
| E4 | MC4 | COI | Umbrina roncador | COI | 3 | 0.00020 |
| E4 | MC4 | COI | Engraulis mordax | COI | 1 | 0.00007 |
| F4 | MC4 | COI | Citharichthys stigmaeus | COI | 48 | 0.00252 |
| F4 | MC4 | COI | Etrumeus teres | COI | 10 | 0.00053 |
| F4 | MC4 | COI | Umbrina roncador | COI | 8 | 0.00042 |
| F4 | MC4 | COI | Citharichthys sordidus | COI | 2 | 0.00011 |
| F4 | MC4 | COI | Cynoscion parvipinnis | COI | 2 | 0.00011 |
| F4 | MC4 | COI | Engraulis mordax | COI | 2 | 0.00011 |
| F4 | MC4 | COI | Paralichthys californicus | COI | 2 | 0.00011 |
| F4 | MC4 | COI | Paralabrax clathratus | COI | 1 | 0.00005 |
| G4 | MC4 | COI | Citharichthys stigmaeus | COI | 72 | 0.00314 |
| G4 | MC4 | COI | Citharichthys sordidus | COI | 18 | 0.00079 |
| G4 | MC4 | COI | Umbrina roncador | COI | 11 | 0.00048 |
| G4 | MC4 | COI | Etrumeus teres | COI | 3 | 0.00013 |
| G4 | MC4 | COI | Oxyjulis californica | COI | 1 | 0.00004 |
| G4 | MC4 | COI | Paralabrax clathratus | COI | 1 | 0.00004 |
| H4 | MC4 | COI | Citharichthys stigmaeus | COI | 64 | 0.00401 |
| H4 | MC4 | COI | Umbrina roncador | COI | 13 | 0.00081 |
| H4 | MC4 | COI | Etrumeus teres | COI | 4 | 0.00025 |
| H4 | MC4 | COI | Cheilotrema saturnum | COI | 2 | 0.00013 |
| H4 | MC4 | COI | Paralichthys californicus | COI | 2 | 0.00013 |
| H4 | MC4 | COI | Citharichthys sordidus | COI | 1 | 0.00006 |
| H4 | MC4 | COI | Oxyjulis californica | COI | 1 | 0.00006 |
| A5 | MC5 | COI | Umbrina roncador | COI | 106 | 0.00829 |
| A5 | MC5 | COI | Citharichthys sordidus | COI | 8 | 0.00063 |
| A5 | MC5 | COI | Etrumeus teres | COI | 2 | 0.00016 |
| A5 | MC5 | COI | Oxyjulis californica | COI | 2 | 0.00016 |
| A5 | MC5 | COI | Seriphus politus | COI | 1 | 0.00008 |
| B5 | MC5 | COI | Umbrina roncador | COI | 17 | 0.00135 |
| B5 | MC5 | COI | Oxyjulis californica | COI | 3 | 0.00024 |
| B5 | MC5 | COI | Cynoscion parvipinnis | COI | 1 | 0.00008 |
| B5 | MC5 | COI | Etrumeus teres | COI | 1 | 0.00008 |
| B5 | MC5 | COI | Paralabrax clathratus | COI | 1 | 0.00008 |
| B5 | MC5 | COI | Seriphus politus | COI | 1 | 0.00008 |
| C5 | MC5 | COI | Etrumeus teres | COI | 2 | 0.00015 |
| C5 | MC5 | COI | Seriphus politus | COI | 1 | 0.00007 |
| D5 | MC5 | COI | Oxyjulis californica | COI | 4 | 0.00016 |
| D5 | MC5 | COI | Umbrina roncador | COI | 3 | 0.00012 |
| D5 | MC5 | COI | Seriphus politus | COI | 2 | 0.00008 |
| D5 | MC5 | COI | Etrumeus teres | COI | 1 | 0.00004 |
| E5 | MC6 | COI | Etrumeus teres | COI | 770 | 0.05308 |
| E5 | MC6 | COI | Halichoeres semicinctus | COI | 556 | 0.03833 |
| E5 | MC6 | COI | Citharichthys stigmaeus | COI | 108 | 0.00744 |
| E5 | MC6 | COI | Citharichthys sordidus | COI | 8 | 0.00055 |
| E5 | MC6 | COI | Oxyjulis californica | COI | 5 | 0.00034 |
| E5 | MC6 | COI | Engraulis mordax | COI | 2 | 0.00014 |
| E5 | MC6 | COI | Umbrina roncador | COI | 1 | 0.00007 |
| F5 | MC6 | COI | Etrumeus teres | COI | 1685 | 0.05519 |
| F5 | MC6 | COI | Halichoeres semicinctus | COI | 1039 | 0.03403 |
| F5 | MC6 | COI | Citharichthys stigmaeus | COI | 234 | 0.00766 |
| F5 | MC6 | COI | Umbrina roncador | COI | 21 | 0.00069 |
| F5 | MC6 | COI | Oxyjulis californica | COI | 15 | 0.00049 |
| F5 | MC6 | COI | Citharichthys sordidus | COI | 6 | 0.00020 |
| F5 | MC6 | COI | Menticirrhus undulatus | COI | 2 | 0.00007 |
| F5 | MC6 | COI | Citharichthys xanthostigma | COI | 1 | 0.00003 |
| G5 | MC6 | COI | Etrumeus teres | COI | 1085 | 0.05094 |
| G5 | MC6 | COI | Halichoeres semicinctus | COI | 648 | 0.03043 |
| G5 | MC6 | COI | Citharichthys stigmaeus | COI | 134 | 0.00629 |
| G5 | MC6 | COI | Oxyjulis californica | COI | 9 | 0.00042 |
| G5 | MC6 | COI | Umbrina roncador | COI | 8 | 0.00038 |
| G5 | MC6 | COI | Engraulis mordax | COI | 5 | 0.00023 |
| G5 | MC6 | COI | Symphurus atricaudus | COI | 2 | 0.00009 |
| G5 | MC6 | COI | Citharichthys sordidus | COI | 1 | 0.00005 |
| G5 | MC6 | COI | Citharichthys xanthostigma | COI | 1 | 0.00005 |
| G5 | MC6 | COI | Menticirrhus undulatus | COI | 1 | 0.00005 |
| H5 | MC6 | COI | Etrumeus teres | COI | 728 | 0.05222 |
| H5 | MC6 | COI | Halichoeres semicinctus | COI | 464 | 0.03328 |
| H5 | MC6 | COI | Citharichthys stigmaeus | COI | 86 | 0.00617 |
| H5 | MC6 | COI | Menticirrhus undulatus | COI | 4 | 0.00029 |
| H5 | MC6 | COI | Oxyjulis californica | COI | 3 | 0.00022 |
| H5 | MC6 | COI | Citharichthys xanthostigma | COI | 1 | 0.00007 |
| H5 | MC6 | COI | Umbrina roncador | COI | 1 | 0.00007 |
| A6 | MC1 | 16S | Sardinops sagax | 16S | 71 | 0.00652 |
| A6 | MC1 | 16S | Semicossyphus pulcher | 16S | 26 | 0.00239 |
| A6 | MC1 | 16S | Scomber japonicus | 16S | 16 | 0.00147 |
| A6 | MC1 | 16S | Anchoa delicatissima | 16S | 7 | 0.00064 |
| A6 | MC1 | 16S | Halichoeres semicinctus | 16S | 2 | 0.00018 |
| A6 | MC1 | 16S | Xenistius californiensis | 16S | 2 | 0.00018 |
| B6 | MC1 | 16S | Sardinops sagax | 16S | 33 | 0.00547 |
| B6 | MC1 | 16S | Scomber japonicus | 16S | 17 | 0.00282 |
| B6 | MC1 | 16S | Semicossyphus pulcher | 16S | 11 | 0.00182 |
| B6 | MC1 | 16S | Anchoa delicatissima | 16S | 1 | 0.00017 |
| B6 | MC1 | 16S | Halichoeres semicinctus | 16S | 1 | 0.00017 |
| C6 | MC1 | 16S | Sardinops sagax | 16S | 135 | 0.00734 |
| C6 | MC1 | 16S | Semicossyphus pulcher | 16S | 50 | 0.00272 |
| C6 | MC1 | 16S | Scomber japonicus | 16S | 35 | 0.00190 |
| C6 | MC1 | 16S | Anchoa delicatissima | 16S | 2 | 0.00011 |
| C6 | MC1 | 16S | Xenistius californiensis | 16S | 2 | 0.00011 |
| C6 | MC1 | 16S | Citharichthys xanthostigma | 16S | 1 | 0.00005 |
| C6 | MC1 | 16S | Halichoeres semicinctus | 16S | 1 | 0.00005 |
| D6 | MC1 | 16S | Sardinops sagax | 16S | 90 | 0.00547 |
| D6 | MC1 | 16S | Semicossyphus pulcher | 16S | 36 | 0.00219 |
| D6 | MC1 | 16S | Scomber japonicus | 16S | 17 | 0.00103 |
| D6 | MC1 | 16S | Halichoeres semicinctus | 16S | 8 | 0.00049 |
| D6 | MC1 | 16S | Xenistius californiensis | 16S | 1 | 0.00006 |
| E6 | MC2 | 16S | Anchoa delicatissima | 16S | 2 | 0.00014 |
| E6 | MC2 | 16S | Citharichthys stigmaeus | 16S | 1 | 0.00007 |
| E6 | MC2 | 16S | Oxyjulis californica | 16S | 1 | 0.00007 |
| F6 | MC2 | 16S | Anchoa delicatissima | 16S | 6 | 0.00040 |
| F6 | MC2 | 16S | Xenistius californiensis | 16S | 2 | 0.00013 |
| G6 | MC2 | 16S | Anchoa delicatissima | 16S | 3 | 0.00019 |
| G6 | MC2 | 16S | Xenistius californiensis | 16S | 1 | 0.00006 |
| H6 | MC2 | 16S | Anchoa delicatissima | 16S | 9 | 0.00068 |
| H6 | MC2 | 16S | Oxyjulis californica | 16S | 2 | 0.00015 |
| A7 | MC3 | 16S | Engraulis mordax | 16S | 862 | 0.06218 |
| A7 | MC3 | 16S | Pleuronichthys ritteri | 16S | 146 | 0.01053 |
| A7 | MC3 | 16S | Semicossyphus pulcher | 16S | 126 | 0.00909 |
| A7 | MC3 | 16S | Citharichthys stigmaeus | 16S | 74 | 0.00534 |
| A7 | MC3 | 16S | Girella nigricans | 16S | 43 | 0.00310 |
| A7 | MC3 | 16S | Scomber japonicus | 16S | 24 | 0.00173 |
| A7 | MC3 | 16S | Sardinops sagax | 16S | 9 | 0.00065 |
| A7 | MC3 | 16S | Pleuronichthys coenosus | 16S | 7 | 0.00050 |
| A7 | MC3 | 16S | Cynoscion parvipinnis | 16S | 5 | 0.00036 |
| A7 | MC3 | 16S | Xenistius californiensis | 16S | 5 | 0.00036 |
| A7 | MC3 | 16S | Etrumeus acuminatus | 16S | 2 | 0.00014 |
| A7 | MC3 | 16S | Trachurus symmetricus | 16S | 2 | 0.00014 |
| A7 | MC3 | 16S | Anchoa delicatissima | 16S | 1 | 0.00007 |
| A7 | MC3 | 16S | Paralabrax clathratus | 16S | 1 | 0.00007 |
| A7 | MC3 | 16S | Paralabrax nebulifer | 16S | 1 | 0.00007 |
| B7 | MC3 | 16S | Engraulis mordax | 16S | 895 | 0.06223 |
| B7 | MC3 | 16S | Pleuronichthys ritteri | 16S | 129 | 0.00897 |
| B7 | MC3 | 16S | Semicossyphus pulcher | 16S | 114 | 0.00793 |
| B7 | MC3 | 16S | Citharichthys stigmaeus | 16S | 50 | 0.00348 |
| B7 | MC3 | 16S | Girella nigricans | 16S | 30 | 0.00209 |
| B7 | MC3 | 16S | Scomber japonicus | 16S | 13 | 0.00090 |
| B7 | MC3 | 16S | Sardinops sagax | 16S | 9 | 0.00063 |
| B7 | MC3 | 16S | Pleuronichthys coenosus | 16S | 7 | 0.00049 |
| B7 | MC3 | 16S | Etrumeus acuminatus | 16S | 1 | 0.00007 |
| C7 | MC3 | 16S | Engraulis mordax | 16S | 941 | 0.06872 |
| C7 | MC3 | 16S | Pleuronichthys ritteri | 16S | 134 | 0.00979 |
| C7 | MC3 | 16S | Semicossyphus pulcher | 16S | 97 | 0.00708 |
| C7 | MC3 | 16S | Citharichthys stigmaeus | 16S | 53 | 0.00387 |
| C7 | MC3 | 16S | Girella nigricans | 16S | 35 | 0.00256 |
| C7 | MC3 | 16S | Scomber japonicus | 16S | 19 | 0.00139 |
| C7 | MC3 | 16S | Sardinops sagax | 16S | 11 | 0.00080 |
| C7 | MC3 | 16S | Pleuronichthys coenosus | 16S | 6 | 0.00044 |
| C7 | MC3 | 16S | Xenistius californiensis | 16S | 2 | 0.00015 |
| D7 | MC3 | 16S | Engraulis mordax | 16S | 6 | 0.07317 |
| D7 | MC3 | 16S | Citharichthys stigmaeus | 16S | 1 | 0.01220 |
| D7 | MC3 | 16S | Pleuronichthys ritteri | 16S | 1 | 0.01220 |
| D7 | MC3 | 16S | Semicossyphus pulcher | 16S | 1 | 0.01220 |
| D7 | MC3 | 16S | Xenistius californiensis | 16S | 1 | 0.01220 |
| E7 | MC4 | 16S | Oxyjulis californica | 16S | 80 | 0.00327 |
| E7 | MC4 | 16S | Hermosilla azurea | 16S | 3 | 0.00012 |
| E7 | MC4 | 16S | Citharichthys stigmaeus | 16S | 2 | 0.00008 |
| E7 | MC4 | 16S | Paralichthys californicus | 16S | 2 | 0.00008 |
| E7 | MC4 | 16S | Cheilotrema saturnum | 16S | 1 | 0.00004 |
| E7 | MC4 | 16S | Engraulis mordax | 16S | 1 | 0.00004 |
| E7 | MC4 | 16S | Sardinops sagax | 16S | 1 | 0.00004 |
| F7 | MC4 | 16S | Oxyjulis californica | 16S | 43 | 0.00342 |
| F7 | MC4 | 16S | Engraulis mordax | 16S | 12 | 0.00095 |
| F7 | MC4 | 16S | Citharichthys stigmaeus | 16S | 2 | 0.00016 |
| F7 | MC4 | 16S | Hermosilla azurea | 16S | 1 | 0.00008 |
| F7 | MC4 | 16S | Paralichthys californicus | 16S | 1 | 0.00008 |
| F7 | MC4 | 16S | Sardinops sagax | 16S | 1 | 0.00008 |
| G7 | MC4 | 16S | Oxyjulis californica | 16S | 45 | 0.00269 |
| G7 | MC4 | 16S | Citharichthys stigmaeus | 16S | 4 | 0.00024 |
| H7 | MC4 | 16S | Oxyjulis californica | 16S | 34 | 0.00185 |
| H7 | MC4 | 16S | Citharichthys stigmaeus | 16S | 2 | 0.00011 |
| H7 | MC4 | 16S | Paralichthys californicus | 16S | 2 | 0.00011 |
| H7 | MC4 | 16S | Engraulis mordax | 16S | 1 | 0.00005 |
| H7 | MC4 | 16S | Sardinops sagax | 16S | 1 | 0.00005 |
| A8 | MC5 | 16S | Anchoa delicatissima | 16S | 5 | 0.00040 |
| A8 | MC5 | 16S | Haemulon flaviguttatum | 16S | 1 | 0.00008 |
| A8 | MC5 | 16S | Hermosilla azurea | 16S | 1 | 0.00008 |
| C8 | MC5 | 16S | Anchoa delicatissima | 16S | 1 | 0.00008 |
| C8 | MC5 | 16S | Oxyjulis californica | 16S | 1 | 0.00008 |
| E8 | MC6 | 16S | Citharichthys stigmaeus | 16S | 130 | 0.02766 |
| E8 | MC6 | 16S | Halichoeres semicinctus | 16S | 20 | 0.00426 |
| E8 | MC6 | 16S | Seriola lalandi | 16S | 10 | 0.00213 |
| E8 | MC6 | 16S | Naucrates ductor | 16S | 3 | 0.00064 |
| E8 | MC6 | 16S | Engraulis mordax | 16S | 2 | 0.00043 |
| E8 | MC6 | 16S | Seriola rivoliana | 16S | 2 | 0.00043 |
| E8 | MC6 | 16S | Citharichthys xanthostigma | 16S | 1 | 0.00021 |
| F8 | MC6 | 16S | Citharichthys stigmaeus | 16S | 206 | 0.02283 |
| F8 | MC6 | 16S | Halichoeres semicinctus | 16S | 45 | 0.00499 |
| F8 | MC6 | 16S | Seriola lalandi | 16S | 10 | 0.00111 |
| F8 | MC6 | 16S | Seriola rivoliana | 16S | 10 | 0.00111 |
| F8 | MC6 | 16S | Naucrates ductor | 16S | 5 | 0.00055 |
| F8 | MC6 | 16S | Citharichthys sordidus | 16S | 3 | 0.00033 |
| F8 | MC6 | 16S | Citharichthys xanthostigma | 16S | 2 | 0.00022 |
| F8 | MC6 | 16S | Oxyjulis californica | 16S | 2 | 0.00022 |
| F8 | MC6 | 16S | Engraulis mordax | 16S | 1 | 0.00011 |
| G8 | MC6 | 16S | Citharichthys stigmaeus | 16S | 342 | 0.02313 |
| G8 | MC6 | 16S | Halichoeres semicinctus | 16S | 53 | 0.00359 |
| G8 | MC6 | 16S | Seriola lalandi | 16S | 27 | 0.00183 |
| G8 | MC6 | 16S | Seriola rivoliana | 16S | 11 | 0.00074 |
| G8 | MC6 | 16S | Naucrates ductor | 16S | 10 | 0.00068 |
| G8 | MC6 | 16S | Haemulon flaviguttatum | 16S | 2 | 0.00014 |
| G8 | MC6 | 16S | Engraulis mordax | 16S | 1 | 0.00007 |
| H8 | MC6 | 16S | Citharichthys stigmaeus | 16S | 370 | 0.02435 |
| H8 | MC6 | 16S | Halichoeres semicinctus | 16S | 48 | 0.00316 |
| H8 | MC6 | 16S | Seriola lalandi | 16S | 18 | 0.00118 |
| H8 | MC6 | 16S | Seriola rivoliana | 16S | 18 | 0.00118 |
| H8 | MC6 | 16S | Citharichthys xanthostigma | 16S | 7 | 0.00046 |
| H8 | MC6 | 16S | Naucrates ductor | 16S | 3 | 0.00020 |
| H8 | MC6 | 16S | Engraulis mordax | 16S | 1 | 0.00007 |
| H8 | MC6 | 16S | Haemulon flaviguttatum | 16S | 1 | 0.00007 |
| H8 | MC6 | 16S | Menticirrhus undulatus | 16S | 1 | 0.00007 |
| A11 | MC1 | M | Sardinops sagax | 16S | 82 | 0.00436 |
| A11 | MC1 | M | Halichoeres semicinctus | COI | 38 | 0.00202 |
| A11 | MC1 | M | Semicossyphus pulcher | 16S | 34 | 0.00181 |
| A11 | MC1 | M | Scomber japonicus | 16S | 26 | 0.00138 |
| A11 | MC1 | M | Semicossyphus pulcher | COI | 11 | 0.00059 |
| A11 | MC1 | M | Anchoa delicatissima | 16S | 8 | 0.00043 |
| A11 | MC1 | M | Halichoeres semicinctus | 16S | 7 | 0.00037 |
| A11 | MC1 | M | Scomber japonicus | COI | 4 | 0.00021 |
| A11 | MC1 | M | Cynoscion parvipinnis | COI | 2 | 0.00011 |
| A11 | MC1 | M | Seriphus politus | COI | 2 | 0.00011 |
| A11 | MC1 | M | Seriphus politus | 16S | 1 | 0.00005 |
| B11 | MC1 | M | Sardinops sagax | 16S | 51 | 0.00401 |
| B11 | MC1 | M | Halichoeres semicinctus | COI | 24 | 0.00189 |
| B11 | MC1 | M | Semicossyphus pulcher | 16S | 22 | 0.00173 |
| B11 | MC1 | M | Scomber japonicus | 16S | 21 | 0.00165 |
| B11 | MC1 | M | Scomber japonicus | COI | 7 | 0.00055 |
| B11 | MC1 | M | Halichoeres semicinctus | 16S | 6 | 0.00047 |
| B11 | MC1 | M | Anchoa delicatissima | 16S | 4 | 0.00031 |
| B11 | MC1 | M | Semicossyphus pulcher | COI | 3 | 0.00024 |
| B11 | MC1 | M | Seriphus politus | 16S | 2 | 0.00016 |
| C11 | MC1 | M | Semicossyphus pulcher | 16S | 73 | 0.01199 |
| C11 | MC1 | M | Sardinops sagax | 16S | 54 | 0.00887 |
| C11 | MC1 | M | Scomber japonicus | 16S | 15 | 0.00246 |
| C11 | MC1 | M | Semicossyphus pulcher | COI | 10 | 0.00164 |
| C11 | MC1 | M | Halichoeres semicinctus | COI | 7 | 0.00115 |
| C11 | MC1 | M | Xenistius californiensis | COI | 3 | 0.00049 |
| C11 | MC1 | M | Halichoeres semicinctus | 16S | 1 | 0.00016 |
| C11 | MC1 | M | Menticirrhus undulatus | 16S | 1 | 0.00016 |
| C11 | MC1 | M | Xenistius californiensis | 16S | 1 | 0.00016 |
| D11 | MC1 | M | Semicossyphus pulcher | 16S | 158 | 0.01161 |
| D11 | MC1 | M | Sardinops sagax | 16S | 100 | 0.00735 |
| D11 | MC1 | M | Scomber japonicus | 16S | 32 | 0.00235 |
| D11 | MC1 | M | Semicossyphus pulcher | COI | 27 | 0.00198 |
| D11 | MC1 | M | Halichoeres semicinctus | COI | 25 | 0.00184 |
| D11 | MC1 | M | Anchoa delicatissima | 16S | 7 | 0.00051 |
| D11 | MC1 | M | Halichoeres semicinctus | 16S | 7 | 0.00051 |
| D11 | MC1 | M | Scomber japonicus | COI | 4 | 0.00029 |
| E11 | MC2 | M | Oxyjulis californica | COI | 2 | 0.00052 |
| E11 | MC2 | M | Anchoa delicatissima | 16S | 1 | 0.00026 |
| E11 | MC2 | M | Oxyjulis californica | 16S | 1 | 0.00026 |
| E11 | MC2 | M | Xenistius californiensis | COI | 1 | 0.00026 |
| F11 | MC2 | M | Anchoa delicatissima | 16S | 3 | 0.00040 |
| F11 | MC2 | M | Citharichthys stigmaeus | COI | 1 | 0.00013 |
| F11 | MC2 | M | Oxyjulis californica | 16S | 1 | 0.00013 |
| G11 | MC2 | M | Oxyjulis californica | COI | 2 | 0.00034 |
| G11 | MC2 | M | Anchoa delicatissima | 16S | 1 | 0.00017 |
| G11 | MC2 | M | Oxyjulis californica | 16S | 1 | 0.00017 |
| H11 | MC2 | M | Anchoa delicatissima | 16S | 16 | 0.00110 |
| H11 | MC2 | M | Citharichthys stigmaeus | COI | 3 | 0.00021 |
| H11 | MC2 | M | Xenistius californiensis | 16S | 2 | 0.00014 |
| H11 | MC2 | M | Ophidion scrippsae | COI | 1 | 0.00007 |
| H11 | MC2 | M | Oxyjulis californica | COI | 1 | 0.00007 |
| H11 | MC2 | M | Sardinops sagax CO | -11 | 1 | 0.00007 |
| A12 | MC3 | M | Engraulis mordax | 16S | 445 | 0.05063 |
| A12 | MC3 | M | Engraulis mordax | COI | 149 | 0.01695 |
| A12 | MC3 | M | Semicossyphus pulcher | 16S | 66 | 0.00751 |
| A12 | MC3 | M | Pleuronichthys ritteri | 16S | 54 | 0.00614 |
| A12 | MC3 | M | Citharichthys stigmaeus | 16S | 37 | 0.00421 |
| A12 | MC3 | M | Citharichthys stigmaeus | COI | 12 | 0.00137 |
| A12 | MC3 | M | Girella nigricans | 16S | 10 | 0.00114 |
| A12 | MC3 | M | Semicossyphus pulcher | COI | 9 | 0.00102 |
| A12 | MC3 | M | Scomber japonicus | 16S | 5 | 0.00057 |
| A12 | MC3 | M | Xenistius californiensis | COI | 5 | 0.00057 |
| A12 | MC3 | M | Sardinops sagax | 16S | 4 | 0.00046 |
| A12 | MC3 | M | Pleuronichthys coenosus | 16S | 3 | 0.00034 |
| A12 | MC3 | M | Xenistius californiensis | 16S | 2 | 0.00023 |
| A12 | MC3 | M | Anchoa delicatissima | 16S | 1 | 0.00011 |
| A12 | MC3 | M | Etrumeus acuminatus | 16S | 1 | 0.00011 |
| A12 | MC3 | M | Paralabrax clathratus | COI | 1 | 0.00011 |
| A12 | MC3 | M | Scomber japonicus | COI | 1 | 0.00011 |
| B12 | MC3 | M | Engraulis mordax | 16S | 269 | 0.04049 |
| B12 | MC3 | M | Engraulis mordax | COI | 99 | 0.01490 |
| B12 | MC3 | M | Pleuronichthys ritteri | 16S | 40 | 0.00602 |
| B12 | MC3 | M | Semicossyphus pulcher | 16S | 33 | 0.00497 |
| B12 | MC3 | M | Citharichthys stigmaeus | 16S | 22 | 0.00331 |
| B12 | MC3 | M | Girella nigricans | 16S | 13 | 0.00196 |
| B12 | MC3 | M | Citharichthys stigmaeus | COI | 7 | 0.00105 |
| B12 | MC3 | M | Semicossyphus pulcher | COI | 5 | 0.00075 |
| B12 | MC3 | M | Xenistius californiensis | COI | 5 | 0.00075 |
| B12 | MC3 | M | Paralabrax clathratus | COI | 1 | 0.00015 |
| B12 | MC3 | M | Pleuronichthys coenosus | 16S | 1 | 0.00015 |
| B12 | MC3 | M | Sardinops sagax | 16S | 1 | 0.00015 |
| B12 | MC3 | M | Umbrina roncador | COI | 1 | 0.00015 |
| C12 | MC3 | M | Engraulis mordax | 16S | 523 | 0.05305 |
| C12 | MC3 | M | Engraulis mordax | COI | 162 | 0.01643 |
| C12 | MC3 | M | Pleuronichthys ritteri | 16S | 64 | 0.00649 |
| C12 | MC3 | M | Semicossyphus pulcher | 16S | 57 | 0.00578 |
| C12 | MC3 | M | Citharichthys stigmaeus | 16S | 50 | 0.00507 |
| C12 | MC3 | M | Xenistius californiensis | COI | 33 | 0.00335 |
| C12 | MC3 | M | Citharichthys stigmaeus | COI | 23 | 0.00233 |
| C12 | MC3 | M | Girella nigricans | 16S | 19 | 0.00193 |
| C12 | MC3 | M | Semicossyphus pulcher | COI | 9 | 0.00091 |
| C12 | MC3 | M | Scomber japonicus | 16S | 3 | 0.00030 |
| C12 | MC3 | M | Atractoscion nobilis | COI | 2 | 0.00020 |
| C12 | MC3 | M | Etrumeus acuminatus | 16S | 2 | 0.00020 |
| C12 | MC3 | M | Girella nigricans | COI | 2 | 0.00020 |
| C12 | MC3 | M | Pleuronichthys coenosus | 16S | 2 | 0.00020 |
| C12 | MC3 | M | Sardinops sagax | 16S | 2 | 0.00020 |
| C12 | MC3 | M | Anisotremus davidsonii | COI | 1 | 0.00010 |
| C12 | MC3 | M | Paralabrax clathratus | COI | 1 | 0.00010 |
| C12 | MC3 | M | Paralabrax nebulifer | COI | 1 | 0.00010 |
| D12 | MC3 | M | Engraulis mordax | 16S | 368 | 0.04290 |
| D12 | MC3 | M | Engraulis mordax | COI | 156 | 0.01818 |
| D12 | MC3 | M | Semicossyphus pulcher | 16S | 51 | 0.00594 |
| D12 | MC3 | M | Pleuronichthys ritteri | 16S | 49 | 0.00571 |
| D12 | MC3 | M | Xenistius californiensis | 16S | 46 | 0.00536 |
| D12 | MC3 | M | Citharichthys stigmaeus | 16S | 39 | 0.00455 |
| D12 | MC3 | M | Sardinops sagax | 16S | 22 | 0.00256 |
| D12 | MC3 | M | Girella nigricans | 16S | 20 | 0.00233 |
| D12 | MC3 | M | Citharichthys stigmaeus | COI | 13 | 0.00152 |
| D12 | MC3 | M | Xenistius californiensis | COI | 6 | 0.00070 |
| D12 | MC3 | M | Pleuronichthys coenosus | 16S | 5 | 0.00058 |
| D12 | MC3 | M | Scomber japonicus | 16S | 5 | 0.00058 |
| D12 | MC3 | M | Semicossyphus pulcher | COI | 4 | 0.00047 |
| D12 | MC3 | M | Umbrina roncador | 16S | 4 | 0.00047 |
| D12 | MC3 | M | Anchoa delicatissima | 16S | 2 | 0.00023 |
| D12 | MC3 | M | Girella nigricans | COI | 2 | 0.00023 |
| D12 | MC3 | M | Cynoscion parvipinnis | COI | 1 | 0.00012 |
| D12 | MC3 | M | Etrumeus teres | COI | 1 | 0.00012 |
| D12 | MC3 | M | Scomber japonicus | COI | 1 | 0.00012 |
| E12 | MC4 | M | Citharichthys stigmaeus | 16S | 50 | 0.00428 |
| E12 | MC4 | M | Engraulis mordax | 16S | 50 | 0.00428 |
| E12 | MC4 | M | Oxyjulis californica | 16S | 21 | 0.00180 |
| E12 | MC4 | M | Engraulis mordax | COI | 18 | 0.00154 |
| E12 | MC4 | M | Paralichthys californicus | 16S | 14 | 0.00120 |
| E12 | MC4 | M | Citharichthys stigmaeus | COI | 8 | 0.00068 |
| E12 | MC4 | M | Oxyjulis californica | COI | 8 | 0.00068 |
| E12 | MC4 | M | Paralichthys californicus | COI | 5 | 0.00043 |
| E12 | MC4 | M | Umbrina roncador | COI | 4 | 0.00034 |
| E12 | MC4 | M | Etrumeus teres | COI | 2 | 0.00017 |
| E12 | MC4 | M | Sardinops sagax | 16S | 2 | 0.00017 |
| E12 | MC4 | M | Umbrina roncador | 16S | 2 | 0.00017 |
| E12 | MC4 | M | Chilara taylori | 16S | 1 | 0.00009 |
| E12 | MC4 | M | Paralabrax clathratus | 16S | 1 | 0.00009 |
| E12 | MC4 | M | Paralabrax maculatofasciatus | 16S | 1 | 0.00009 |
| E12 | MC4 | M | Semicossyphus pulcher | 16S | 1 | 0.00009 |
| F12 | MC4 | M | Oxyjulis californica | 16S | 14 | 0.00178 |
| F12 | MC4 | M | Citharichthys stigmaeus | COI | 6 | 0.00076 |
| F12 | MC4 | M | Oxyjulis californica | COI | 4 | 0.00051 |
| F12 | MC4 | M | Sardinops sagax | 16S | 2 | 0.00025 |
| F12 | MC4 | M | Anisotremus davidsonii | COI | 1 | 0.00013 |
| F12 | MC4 | M | Citharichthys stigmaeus | 16S | 1 | 0.00013 |
| F12 | MC4 | M | Engraulis mordax | 16S | 1 | 0.00013 |
| G12 | MC4 | M | Oxyjulis californica | 16S | 17 | 0.00184 |
| G12 | MC4 | M | Citharichthys stigmaeus | 16S | 15 | 0.00162 |
| G12 | MC4 | M | Citharichthys stigmaeus | COI | 13 | 0.00141 |
| G12 | MC4 | M | Oxyjulis californica | COI | 9 | 0.00097 |
| G12 | MC4 | M | Umbrina roncador | 16S | 5 | 0.00054 |
| G12 | MC4 | M | Sardinops sagax | 16S | 4 | 0.00043 |
| G12 | MC4 | M | Engraulis mordax | 16S | 2 | 0.00022 |
| G12 | MC4 | M | Engraulis mordax | COI | 2 | 0.00022 |
| G12 | MC4 | M | Paralabrax clathratus | 16S | 2 | 0.00022 |
| G12 | MC4 | M | Umbrina roncador | COI | 2 | 0.00022 |
| G12 | MC4 | M | Anisotremus davidsonii | COI | 1 | 0.00011 |
| G12 | MC4 | M | Cheilotrema saturnum | 16S | 1 | 0.00011 |
| G12 | MC4 | M | Etrumeus teres | COI | 1 | 0.00011 |
| G12 | MC4 | M | Paralichthys californicus | 16S | 1 | 0.00011 |
| G12 | MC4 | M | Sphyraena argentea | 16S | 1 | 0.00011 |
| H12 | MC4 | M | Citharichthys stigmaeus | 16S | 38 | 0.00641 |
| H12 | MC4 | M | Engraulis mordax | 16S | 16 | 0.00270 |
| H12 | MC4 | M | Sardinops sagax | 16S | 16 | 0.00270 |
| H12 | MC4 | M | Oxyjulis californica | 16S | 10 | 0.00169 |
| H12 | MC4 | M | Umbrina roncador | 16S | 5 | 0.00084 |
| H12 | MC4 | M | Engraulis mordax | COI | 4 | 0.00067 |
| H12 | MC4 | M | Oxyjulis californica | COI | 4 | 0.00067 |
| H12 | MC4 | M | Citharichthys stigmaeus | COI | 3 | 0.00051 |
| H12 | MC4 | M | Etrumeus teres | COI | 2 | 0.00034 |
| H12 | MC4 | M | Paralichthys californicus | 16S | 2 | 0.00034 |
| H12 | MC4 | M | Scomber japonicus | 16S | 2 | 0.00034 |
| H12 | MC4 | M | Semicossyphus pulcher | 16S | 2 | 0.00034 |
| H12 | MC4 | M | Anisotremus davidsonii | 16S | 1 | 0.00017 |
| H12 | MC4 | M | Cheilotrema saturnum | 16S | 1 | 0.00017 |
| H12 | MC4 | M | Paralabrax clathratus | COI | 1 | 0.00017 |
| H12 | MC4 | M | Paralabrax nebulifer | 16S | 1 | 0.00017 |
| H12 | MC4 | M | Umbrina roncador | COI | 1 | 0.00017 |
